# Conversion of a telomere resolvase into a Cre-like site-specific recombinase

**DOI:** 10.1371/journal.pone.0328478

**Published:** 2025-07-17

**Authors:** Shu Hui Huang, Mahrokh Balouchi, Kerri Kobryn

**Affiliations:** Department of Biochemistry, Microbiology & Immunology, College of Medicine, University of Saskatchewan, Saskatoon, Saskatchewan, Canada; Tulane University Health Sciences Center, UNITED STATES OF AMERICA

## Abstract

Hairpin telomere resolvases are a unique family of enzymes involved in producing the hairpin (hp) telomeres of bacterial organisms and phages that possess linear DNA’s terminated by hp telomeres. The hp telomeres help to overcome the end-replication problem faced by linear DNAs and are generated from replicated intermediates of the linear DNAs. The telomere resolvases employ a reaction mechanism and catalytic domain related to that of the type IB topoisomerases and tyrosine recombinases. ResT, the telomere resolvase from *Borrelia burgdorferi,* under certain reaction conditions, has been shown to promote site-specific recombination between replicated telomere junctions (*rTel*s) to produce a Holliday junction intermediate in a reaction strikingly similar to that promoted by tyrosine recombinases. TelA, the telomere resolvase of *Agrobacterium tumefaciens,* has been shown to be autoinhibited in such a manner as to forbid recombination between *rTel*s. Relief of such autoinhibition reveals a weak, cryptic recombination activity in TelA. In the present study we characterize a catalytic domain aspartic acid residue mutation (D398A) that produces an enzyme with compromised telomere resolution activity but a massively stimulated ability to promote recombination between replicated telomere junctions to produce both the Holliday junction intermediate and full recombinant products of site-specific recombination between *rTel*s. We also report that combination of the D398A mutation with previously characterized hyperactivating mutations in TelA produced a complete conversion of a telomere resolvase into a site-specific recombinase. The possible utility of this conversion is explored.

## Introduction

**Fig 1:**
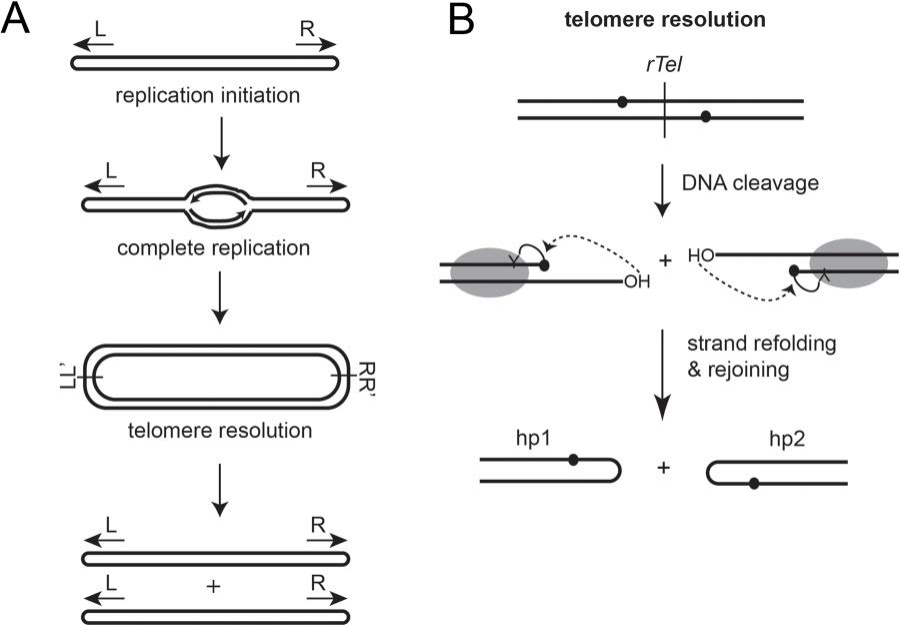
Replication and resolution pathways for linear hairpin replicons in *Borrelia* and *Agrobacterium.* A) Replication initiation initiates at an internal *oriC* sending two replication forks out from the origin towards the hairpin (hp) telomeres. Replication around the hp turnarounds completes replication to produce a circular inverted repeat replicon dimer that is then resolved into two linear DNA’s terminated by hp telomeres by a DNA cleavage and rejoining reaction referred to as telomere resolution. B) Telomere resolution promoted by telomere resolvases. Telomere resolvases dimerize on a replicated telomere junction (*rTel*) and utilize an active site tyrosine (Y) as a nucleophile for cleavage of the scissile phosphates. DNA cleavage produces a transient cleavage intermediate with the telomere resolvase covalently linked to the cleaved strands via 3’-phosphotyrosine linkages. The strands are folded into a hairpin conformation and the 5’-OH’s attack the 3’-phosphotyrosine bonds to reseal the DNA into a pair of hp telomeres.

Telomere resolvases are a unique family of DNA cleavage and rejoining enzymes that create the hairpin (hp) telomeres that terminate the linear replicons of bacteria in the *Borrelia* and *Agrobacterium* genera. The reaction they promote is called telomere resolution and involves the cleavage of short, inverted repeat recognitions sites, referred to as replicated telomeres (*rTels*) within replicated intermediates, followed by the rearrangement of the cleaved DNA strands into a hairpin conformation. Telomere resolution is completed when the cleaved and rearranged strands are resealed (ref. [1] and **Fig 1**). Telomere resolvases employ a reaction mechanism similar to that of the type-IB topoisomerases and the tyrosine recombinase families of enzymes [2–5]. Telomere resolvases share with these two enzyme families a structurally similar catalytic domain that employs a nucleophilic tyrosine residue and a conserved constellation of active site residues that stabilize the transition state and that act as general acid/bases to activate the tyrosine and DNA nucleophiles and to protonate the DNA leaving group. The reaction is characterized by DNA cleavage executed by the activated tyrosine nucleophiles producing 3’-phosphotyrosine linkages and 5’-OH leaving groups on the cleaved strands. The telomere resolvases then use various mechanisms to refold the cleaved strands into a hairpin (hp) conformation; this positions the 5’-OH ends for nucleophilic attack on the 3’-phosphotyrosine linkages resulting in resealing the cleaved strands into hp telomeres (**Fig 1B**). Telomere resolvases dimerize on their *rTel* substrate DNAs and act as dimers when promoting the reaction [1].

The type-IB topoisomerases and tyrosine recombinases follow an essentially identical chemical scheme for their reactions but produce very different reaction products. Type-IB topoisomerases, typified by vaccinia virus topoisomerase I, catalyze the relaxation of DNA supercoils by cleavage of a single DNA strand followed, after rotation of the cleaved strand, by strand resealing to produce a product with a linking change of 1 [6]. Type-IB topoisomerases, consequently, act as monomers. Tyrosine recombinases, typified by Cre from phage P1, cleave and exchange four strands between two small recognition sites to produce integration, deletion or inversion products depending upon the disposition of the recognition sites on the recombining DNA. Tyrosine recombinases assemble on their reaction sites (dimerize) and then synapse the reaction sites together (tetramerize). A DNA strand in each recognition site is cleaved, the strands are exchanged to produce a Holliday junction (HJ) and then the HJ is subtly isomerized to activate the second pair of strands for cleavage and exchange to resolve the HJ into recombinants [7]. As four DNA strands are cleaved and exchanged tyrosine recombinases act as tetramers. Telomere resolvases fall half-way in this continuum between type-IB topoisomerases and tyrosine recombinases; cleaving and exchanging two DNA strands to produce hp telomeres by assembling into and acting as dimers [8].

Two members of the telomere resolvase family, ResT from *Borellia burgdorferi* and TelA from *Agrobacterium tumefaciens*, have been shown to possess latent tyrosine recombinase activity. This has been shown by the ability of these enzymes, if presented with altered reaction conditions, mutated substrates or via mutation of the enzymes themselves, to promote HJ junction formation between a pair of *rTels* rather than resolving the two *rTels* into hp telomeres. For ResT this activity was discovered when ResT was reacted with a negatively supercoiled plasmid harbouring two *rTels*. It was further discovered that it could also form HJ’s by strand exchange between an *rTel* on a negatively supercoiled plasmid substrate and a small, linear synthetic *rTel* [9]. This alternate, competing side reaction of recombination was observed when at least one reacting partner was on a negatively supercoiled plasmid; negative supercoiling inhibits the normal reaction of telomere resolution [9]. Interestingly, positive supercoiling stimulates the telomere resolution reaction and inhibits HJ formation in the same assay pairing a plasmid substrate and small synthetic *rTel* [9,10]. TelA has also been shown to possess a latent recombinase activity when the autoinhibitory N-terminal domain is deleted or when certain combinations of hyperactivating mutations are present in TelA [11,12]. Both ResT and TelA have also been observed to act as a topoisomerase relaxing plasmids harbouring an *rTel* with an asymmetrized sequence between the scissile phosphates that inhibits hairpin formation [9,12,13].

In the present study we report a TelA mutational study that has uncovered in greater detail the relationship of telomere resolvases to the tyrosine recombinases. We have done this by uncovering mutations and combinations of mutations that suppress telomere resolution and promote site-specific recombination to the extent we report the complete conversion of TelA from a telomere resolvase into site-specific recombinase.

## Materials and methods

### DNAs

All synthetic DNAs used to assemble substrates or produce site-directed mutations were purchased from Integrated DNA Technologies (IDT) and are listed in S1 Table in S1 File. Telomere resolution and recombination assays primarily used the pEKK494 or pEKK495 plasmid substrates; the construction of these plasmids is reported elsewhere [11,14]. The plasmid with the asymmetric *rTel* sequence (CATTGA) between the scissile phosphates, designated as pEKK592, was constructed by annealing OKBA69/70 oligos followed by BamHI-HindIII directional cloning into pUC19 as described for the construction of pEKK494 (TCATGA) and pEKK495 (CCATGA). The telomeric and mock telomeric half-site plasmids (pEKK588 and pEKK589) were constructed by annealing OKBA61/62 and OKBA63/64 oligos, respectively, followed by BamHI-HindIII directional cloning into pUC19 as described above. The mock *rTel* plasmid (pEKK590) was constructed by annealing OKBA65/66 followed by directional cloning.

### Proteins

The TelA mutants reported in this study were generated by site-directed mutagenesis using the oligonucleotides listed in **S1 Table** in S1 File. The induction and expression conditions are listed in S2 Table in S1 File. All TelA purifications proceeded as reported elsewhere [11,15]. SspI, XhoI and T7 endonuclease I were sourced from New England Biolabs.

### TelA plasmid-based telomere resolution and recombination assays

Telomere resolution and recombination assays were incubated at 30^o^C. The reaction buffer contained 25 mM HEPES (pH 7.6), 1 mM DTT, 100 μg/mL BSA, 50 mM potassium glutamate, 2 μg/mL of the noted plasmid substrate DNA, 50 nM TelA and 2 mM MgCl_2_. A 100 μL reaction volume was used and the reaction was terminated by addition of 5X SDS-containing load dye to a 1X concentration. 1X load dye contains 0.2% SDS, 20 mM EDTA, 3.2% glycerol, and 0.024% bromophenol blue. Subsequently, the samples were loaded to 0.8% agarose 1X TAE gels that were electrophoresed at 1V/cm for 21 hours. The results were visualized by staining the gels with 0.5 μg/mL ethidium bromide for 30 min, followed by destaining in distilled water for 30 min. Gel images were visualized using a BioRad GelDoc system. When timecourses were conducted a 100 μL reaction volume was used and 18 μL aliquots were removed and the reaction terminated by addition of 5X SDS-containing load dye to a 1X concentration.

Where the products of recombination were analyzed using restriction digest and treatment with T7 endonuclease I reactions were heat killed at 65^o^C for 20 min prior to adjusting the MgCl_2_ concentration to 10 mM followed by the addition of 2.5 units of SspI (37^o^C, 1 h incubation). Where indicated there was also subsequent addition of 3 units of T7 endonuclease I (30^o^C, 10 min incubation). The samples were then loaded to 0.8% agarose 1X TAE gels that were electrophoresed at 3V/cm for 3 hours.

### Gel-purification of putative recombinant dimers and Holliday junctions

Concentrated recombination reactions were performed in the standard buffer noted above using 10 μg/mL plasmid and a 125 nM concentration of the noted TelA mutants (5X concentrations of substrate DNA and TelA relative to reactions not used for gel purification). For the isolation of SspI-digested HJ forms the reaction was heat killed at 65^o^C for 20 min then supplemented with MgCl_2_ to a final concentration of 10 mM and treated with 2.5 units of SspI at 37^o^C for 1 hour. Putative recombinant dimers (supercoiled) and HJs (SspI-digested) were isolated from an ethidium bromide stained 0.8% agarose 1X TAE preparative gel by visualizing the DNA on a 365 nm transilluminator. The gel slices (~400 mg) were placed in Corning’s Costar Spin-X (R) columns and frozen for 30 minutes. The Spin-X columns were then spun in a microfuge at high speed for 10 min at room temperature and the volume of the eluates determined. The filters that trap the remnant agarose were discarded and to the eluates was added a 1/10th volume of 3M sodium acetate (pH 5.2) and 2.5 volumes of ice cold 95% ethanol followed by vigorous agitation. The ethanol precipitations were allowed to proceed at -20^o^C overnight followed by a high-speed spin in a microfuge (at 4^o^C) for 30 min. The resulting pellet was washed with 1 volume of 70% cold ethanol and spun at high speed (at 4^o^C) for 10 min. After removal of the ethanol the samples were air dried for 10 min at room temperature and the isolated DNAs were resuspended in 40 μL of HE buffer. The resulting putative recombinant dimers and SspI-digested HJs were resuspended in a buffer containing 25 mM HEPES (pH 7.6), 1 mM DTT, 100 μg/mL BSA, 10 mM MgCl_2_ and 50 mM potassium glutamate. The putative recombinant dimers were treated with SspI (2.5 units 37^o^C 1 h) followed by with 3 units of T7 endonuclease I (30^o^C 10 min). SspI-digested and gel purified HJs were treated with 3 units of T7 endonuclease I at 30^o^C, for the time indicated in the legends, prior to gel loading.

### Telomere resolution and recombination assays with ResT

Telomere resolution and recombination assays were incubated at 30^o^C. The reaction buffer contained 25 mM Tris-HCl (pH 8.5), 1 mM EDTA, 100 μg/mL BSA, 100 mM NaCl, 2 μg/mL of the noted plasmid substrate DNA, and 150 nM ResT. A 60 μL reaction volume was used and the reaction was terminated by addition of 5X SDS-containing load dye to a 1X concentration. When restriction digestion products of recombination were analyzed the samples were loaded to 0.7% agarose 1X TAE gels that were electrophoresed at 3.25 V/cm for 4.5 hours. Where the products of recombination were analyzed using restriction digest the buffer was changed by application of the reactions to G50 spin columns and the buffer conditions were adjusted to 1X NEB2 buffer prior to addition of 10 units of XhoI (37^o^C, 1 h incubation). 1X NEB2 buffer contains 50 mM NaCl, 10 mM Tris-HCl, 10 mM MgCl2, 1 mM DTT, pH 7.9@25°C. Where indicated samples treated were treated with 100 μg/mL pronase (37^o^C, 5 min) prior to gel loading. The results were visualized by staining the gels with 0.5 μg/mL ethidium bromide for 30 min, followed by destaining in distilled water for 30 min. Gel images were visualized using a BioRad GelDoc system.

## Results and discussion

### The TelA (D398A) mutation suppresses telomere resolution and promotes recombination between replicated telomeres (*rTels*)

**Fig 2:**
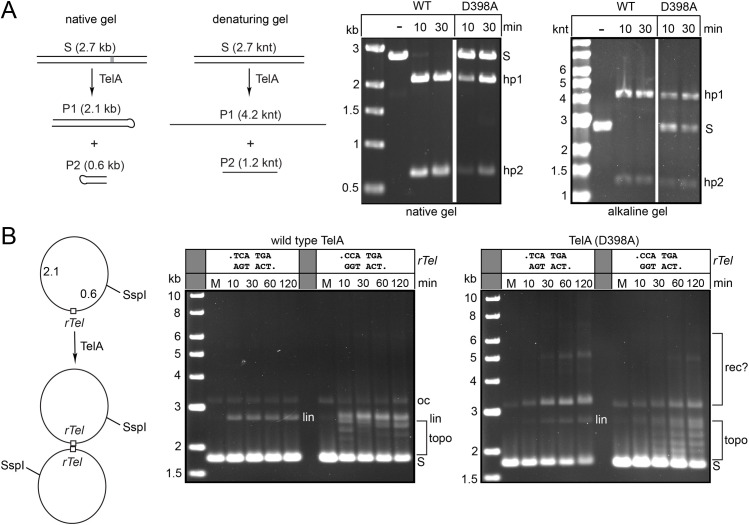
TelA (D398A) promotes both telomere resolution and plasmid multimerization. A) Left: schematic of the telomere resolution reaction visualized on native *vs*. alkaline (denaturing) conditions. Right: The 0.8% agarose 1X TAE (native) and 0.8% agarose alkaline gel panels presented are of telomere resolution reactions conducted with wild type TelA and TelA (D398A) with SspI-linearized plasmid substrate. S denotes substrate while hp1 and hp2 denote the hairpin telomere products of resolution. B) Left: schematic of a recombination reaction with supercoiled plasmid substrate. Right: 0.8% agarose 1X TAE gel panels of reactions with wild type TelA and TelA (D398A) with negatively supercoiled plasmid substrates with wild type sequence between the scissile phosphates (TCATGA; pEKK494) *vs*. a mutant sequence (CCATGA; pEKK495) that suppresses hairpin formation due to an interruption of the dyad symmetry of the wild type sequence at the first nucleotide after the scissile phosphates. S denotes substrate; lin denotes linear DNA; oc denotes open circular DNA; topo denotes topoisomers and rec? denotes probable multimer products of recombination (HJs and full recombinants).

Previous work investigating the mechanism of telomere resolution identified a network of ResT and TelA residues implicated in forming and/or stabilizing an underwound pre-cleavage intermediate that allowed DNA cleavage to occur followed by strand ejection to facilitate hairpin formation and the forward trajectory of the telomere resolution reaction [14,16]. TelA (D398) was one of the residues, in the catalytic domain, that was identified as helping contribute to the forward trajectory of the reaction, probably by helping to underwind the DNA and to eject the strand after DNA cleavage, since the D398A mutant was shown to be cleavage competent but locked into abortive cycles of DNA cleavage and strand rejoining; telomere resolution of this mutant was readily rescued by substrate modifications that mimic ‘pre-melting’ the DNA between the scissile phosphates of the substrate [13,14]. This contention is supported by the close proximity of D398 to the scissile phosphates of the product hairpin telomeres (**S1 Fig** in S1 File). During the course of the examination our suite of TelA mutants for significant differences in reactivity on synthetic substrates assembled from oligonucleotides *vs*. substrate *rTels* cloned into a plasmid we discovered the the TelA (D398A) mutant was significantly less compromised on the plasmid substrates than on the small synthetic substrates used in the original study ( [13] and **Fig 2A; right panel**). While investigating the properties of the TelA (D398A) mutant on plasmid substrates we discovered that with a wild type *rTel* present in a negatively supercoiled plasmid TelA (D398A) readily formed what appeared to be a ladder of plasmid multimers ( [13] and **Fig 2B; right panel**). Reaction with a plasmid substrate harbouring a mutant *rTel* that asymmetrizes the sequence between the scissile phosphates, thereby inhibiting hairpin formation, revealed a cycle of DNA cleavage and rejoining that progressively removes supercoils from the plasmid, yielding a ladder of topoisomers (**Fig 2B; right panel**). The production of the plasmid multimers with supercoiled substrate showed a more marked dependence upon the presence of divalent metal ions than that seen for telomere resolution. There was a preference for Mg^2+^ over Ca^2+^ for multimer production (**S2 Fig** in S1 File).

### Linking D398A to mutations that hyperactivate TelA for telomere resolution switches TelA from a telomere resolvase to a Cre-like recombinase

**Fig 3:**
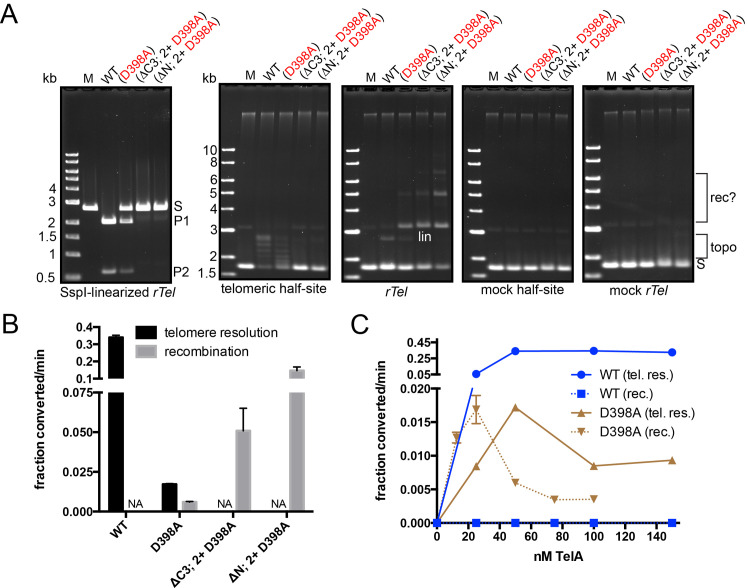
Combining the D398A mutation with mutations that hyperactivate TelA for telomere resolution. A) 0.8% agarose 1X TAE gel panels of incubations of 50 nM of the noted TelA mutants *vs.* wild type TelA using 2 μg/ml of pUC19 variants with telomeric half-site and *rTel* inserts. Incubations with wild type TelA and the D398A mutant were at 30^o^C for 30 min, while incubations with the (ΔC3; D202RE337KD398A) and (ΔN; D202RE337KD398A) mutants were at 30^o^C for 10 min. Additionally, control reactions with mock half-site and mock *rTel* inserts are shown. The half-sites comprise one copy of the symmetry element that makes up the inverted repeat of the *rTel* and mock *rTel* junctions. The mock versions systematically substitute G for C and A for T (and *vice versa*) maintaining the sequence composition of the *bona fide rTel* junction. S denotes substrate; rec? denotes multimers derived from probable recombination of the plasmids; lin (labeled on the *rTel* panel) denotes the position that the linear product of telomere resolution; topo denotes a ladder of topoisomers; P1 & P2 denote the products of telomere resolution with the SspI-linearized *rTel* plasmid. The activating mutations D202RE337K are shortened to (2+) in the gel labels. ΔC3 denotes the deletion of the last three amino acids from the TelA C-terminus; ΔN denotes the deletion of the N-terminal domain (the first 106 amino acids) of TelA. B) Summary graph of the initial rates of telomere resolution *vs.* recombination assayed with 50 nM of the tested TelA variants. NA stands for no activity. C) Summary graph of the initial rates of telomere resolution (tel. res.) *vs.* recombination (rec.) of wild type TelA and the D398A mutant plotted against enzyme concentration. Shown are the mean and standard deviation of three independent trials of each experiment.

In a recent study on TelA we reported a collection of mutants that led to activation of TelA to promote faster telomere resolution reactions by introduction of mutations that reduce the amount of autoinhibition TelA is subject to or increase their affinity for substrate DNA. Some of these mutations, when combined together, produced hyperactivated versions of TelA, often with little dependence upon the presence of divalent metal ions for full reactivity (hyperactive is defined as >50-fold stimulation over wild type TelA; [12]). We were interested to discover what would happen when the D398A mutation was linked to hyperactivating mutations that promote faster telomere resolution. We expected both telomere resolution and recombination to be stimulated in these combined mutant backgrounds. However, what we discovered was that combining D398A with these hyperactivating mutations lead to an almost complete switch in activity from telomere resolution to recombination between *rTels* (**Fig 3A**). Telomere resolution activity was assessed with SspI-linearized *rTel* plasmid while recombination was assessed with a negatively supercoiled version of the same plasmid. The sequence specificity of the recombination reaction to an *rTel* was established by the lack of recombination with a mock *rTel* control plasmid. The requirement for a full-length replicated telomere (*rTel*) site was separately established by control reactions with telomeric and mock telomeric half-site plasmids (**Fig 3A**). As expected, wild type TelA was unable to promote recombination with the supercoiled *rTel* plasmid due to autoinhibition by the combined actions of the N-terminal domain and the C-terminal α-helix [12]. Interestingly, wild type TelA and TelA (D398A) were active as topoisomerases on the telomeric half-site plasmid while the hyperactive recombinase mutants did not relax this or any other tested plasmid. This suggests that wild type TelA and the D398A mutant may be able to act as monomers to bind, cleave and rejoin DNA at the telomeric half-site. The topoisomerase activity was sequence specific since this was not observed with either of the mock telomere substrates (**Fig 3A**). An examination of the divalent metal ion responsiveness of recombination was conducted with all the mutants and showed that only D398A actively required the presence of divalent metal ions to promote recombination (**S2-S4 Figs** in S1 File). This result was expected as the hyperactivating mutations that D398A was linked to in the other mutants have a similar effect on telomere resolution since a principle function of autoinhibition of TelA seems to be to render the telomere resolution reaction responsive to divalent metal ions [11,12].

An examination of the initial rates of the telomere resolution *vs.* recombination reactions with wild type TelA and the mutants underscores the detrimental effect of the D398A mutation on the rate of telomere resolution while allowing a slow rate of recombination absent for wild type TelA (**Fig 3B**). Also clear is the strong stimulatory effect of combining the D398A mutation with the hyperactivating effect of the (ΔC3; D202RE337K) and (ΔN; D202RE337K) mutations on the rate of recombination. The resulting combined mutants (ΔC3; D202RE337KD398A) and (ΔN; D202RE337KD398A) when compared to the (D398A) mutant showed 8.5-fold and 24.5- fold faster initial rates, respectively (**Fig 3B**). Despite this marked stimulation of recombination neither (ΔC3; D202RE337KD398A) nor (ΔN; D202RE337KD398A) showed any detectable activity for telomere resolution (**Fig 3B**).

Because the (D398A) mutant was found to be capable of promoting both telomere resolution and recombination we were interested in determining the TelA concentration optima for these two reactions. The initial rate of reaction for telomere resolution *vs.* recombination was determined for wild type TelA and the (D398A) mutant against a range of TelA concentrations. Wild type TelA displayed no ability to promote recombination over a wide range of TelA concentrations (25–150 nM) while telomere resolution initial rate is maxed out and remains constant at TelA concentrations equal to or greater than 50 nM (**Fig 3C**). While the (D398A) mutant promotes much slower reactions, both telomere resolution and recombination rates displayed equivalent maximal initial rates but at differing TelA concentration optima. The (D398A) mutant showed lower initial rates of reaction either below or above the optimal TelA concentration (**Fig 3C**). Despite, presumably, requiring the synapsis of recombining plasmids, the TelA optimum for recombination promoted by (D398A) was found to be lower than for telomere resolution (25 nM *vs.* 50 nM). These results hinted at possible differences from wild type TelA in the ability of the (D398A) mutant to promote either recombination productive synapsis of *rTel*s or in the oligomerization dynamics of the proteins.

A similar analysis of the initial rate of recombination against TelA concentration conducted with the (ΔC3; D202RE337KD398A) and (ΔN; D202RE337KD398A) mutants revealed that both mutants showed a maximal rate at 50 nM. Neither mutant was active for telomere resolution over the tested concentration range (**S5 Fig** in S1 File).

### Recombination by the TelA (D398A)-containing mutants is accompanied by accumulation of Holliday Junctions (HJs)

**Fig 4:**
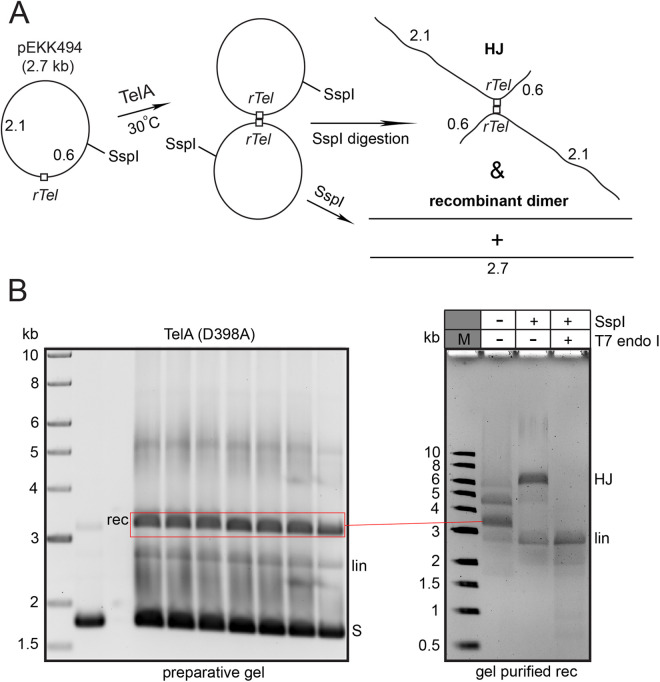
The majority product of recombination by TelA (D398A) is a Holliday junction intermediate. A) Schematic summary of the recombination reaction and the products expected from an SspI-digested recombinant dimer or HJ intermediate. B) 0.8% agarose 1X TAE gel panels of reactions of TelA (D398A) and negatively supercoiled pEKK494 substrate plasmid incubated at 30^o^C for 1h examined by SspI digestion and treatment of the SspI-digested products with the HJ-specific T7 endonuclease I enzyme. The left panel shows the preparative gel used to excise the recombinant dimer form. The bands boxed in red were excised and the DNA purified from the gel as noted in the Materials and methods section. The right panel presents the results with a gel-purified putative recombinant dimer (boxed in red in the first panel). S denotes the supercoiled substrate; lin denotes the linear product of telomere resolution in the preparative gel and the product of SspI digestion of the recombinant dimer in the analysis gel; rec denotes the putative recombinant dimer/HJ mix; HJ denotes the Holliday junction form that has been digested with SspI. The ethidium bromide-stained gels are shown as inverted images.

**Fig 5:**
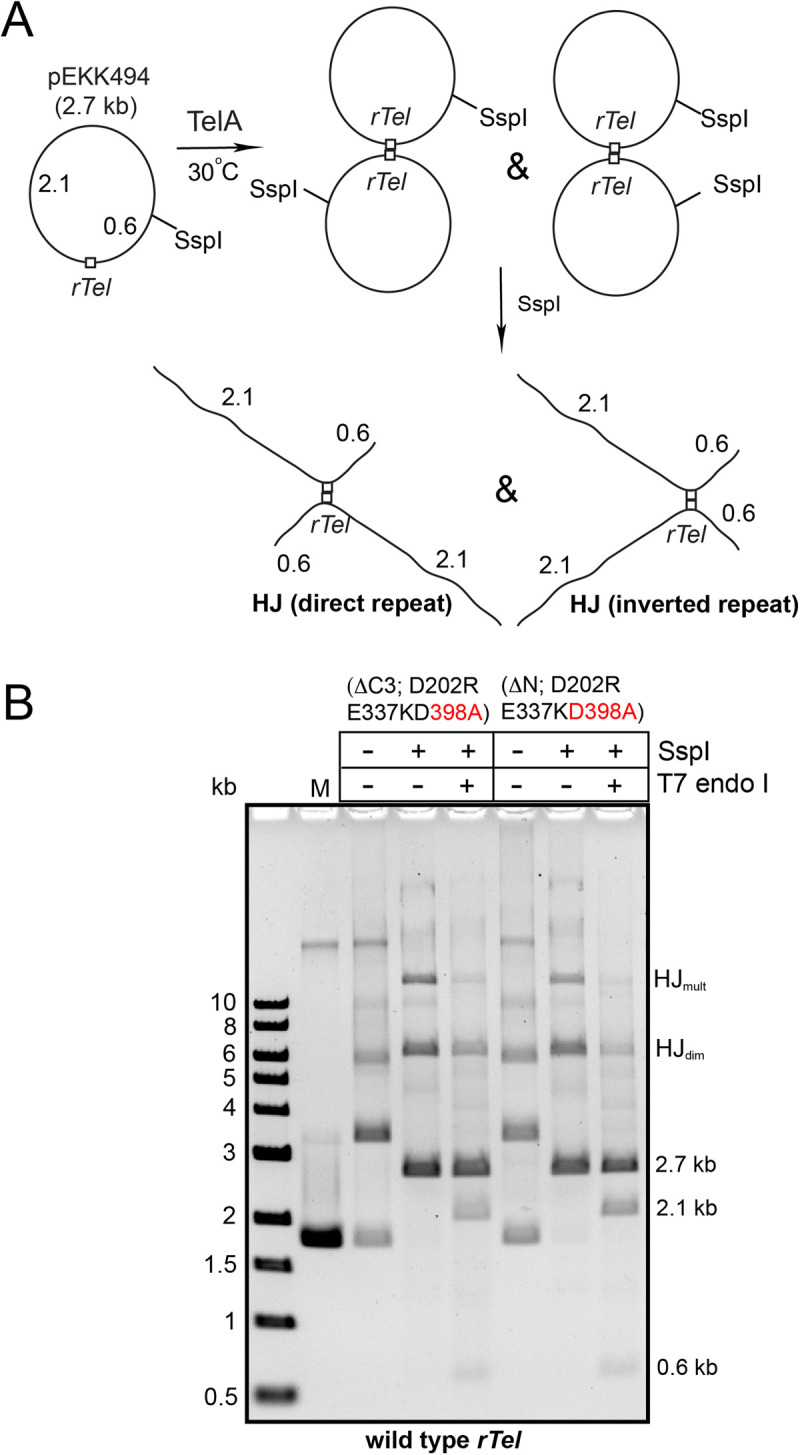
The predominant product of recombination by the (ΔC3; D202RE337KD398A) and (ΔN; D202RE337KD398A) mutants are Holliday junction intermediates. A) Schematic summary of the recombination reaction and the products expected from an SspI-digested HJ intermediate. B) 0.8% agarose 1X TAE gel panel of reactions of the (ΔC3; D202RE337KD398A) and (ΔN; D202RE337KD398A) mutants with negatively supercoiled pEKK494 (wild type *rTel*) substrate plasmid examined by SspI digestion and treatment of the SspI-digested products with the HJ-specific T7 endonuclease I enzyme. The incubations were conducted at 30^o^C for 60 min and 30 min, respectively, for the two mutants. HJ_dim_ denotes a HJ from synapsis and strand exchange between two monomers; HJ_mult_ denotes the presumptive product resulting from synapsis and strand exchange between full recombinants to produce a product with two HJs. The ethidium bromide-stained gels are shown as inverted images.

**Fig 6:**
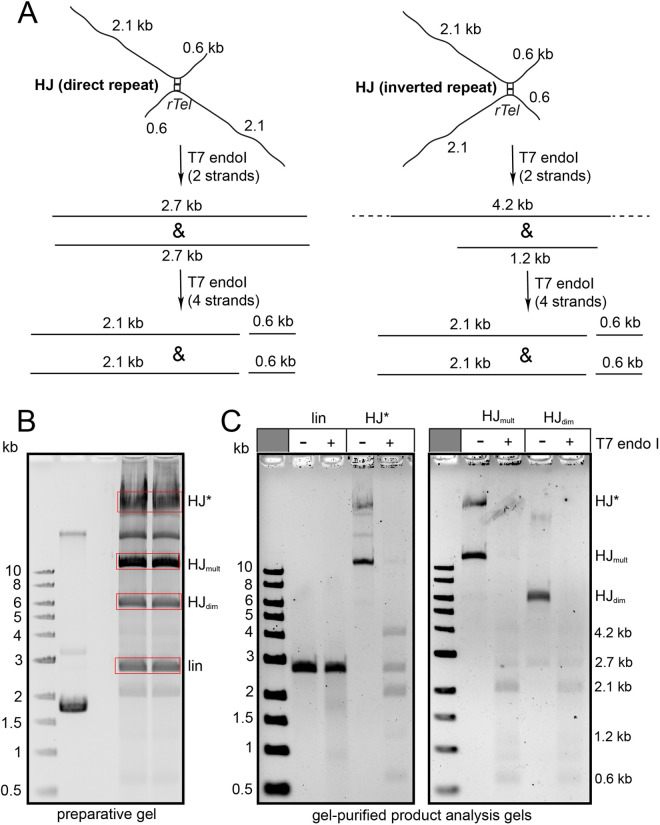
Analysis of gel-purified Holliday junctions produced by a hyperactive recombinase mutant. A) Schematic of the hypothesized structure of the HJs resulting from alternate orientations of synapsis and strand exchange. Also shown are the expected results from incubation with a HJ resolvase (T7 endo I) in the case where two strands or all four strands of the HJ are cleaved. B) 0.8% agarose 1X TAE gel panel of a concentrated reaction of the (ΔC3; D202RE337KD398A) mutant with wild type *rTel* (pEKK494) using a 30^o^C 2 h incubation followed by digestion of the reaction with SspI for isolation of the various forms present in the preparative gel. The bands boxed in red were excised and the DNA purified from the gel as noted in the Materials and methods section. C) Shown are 0.8% agarose 1X TAE gels of the noted gel-purified forms analyzed by digestion with the HJ resolving enzyme T7 endonuclease I. lin denotes SspI-linearized substrate DNA; HJ_dimer_ denotes the presumptive HJ resulting from synapsis and strand exchange between monomers to produce a HJ for a dimer; HJ_mult_ denotes the presumptive product resulting from synapsis and strand exchange between full recombinants to produce a product with two HJs; HJ* denotes the presumptive products resulting from strand exchange between higher order recombinants to produce very large products connected by multiple HJs. The ethidium bromide-stained gels are shown as inverted images.

Previous findings with ResT that demonstrated that, under certain conditions, the *Borrelia burgdorferi* telomere resolvase, ResT, could promote site-specific recombination between *rTels* to the stage of a HJ [9]. We hypothesized that much of the apparent plasmid dimer form produced by the mutants would be the result of a similar reaction with TelA that accumulates the HJ intermediate of recombination. The apparent plasmid dimer produced by reaction with TelA (D398A) was gel-purified and then digested with the single-hit restriction enzyme SspI to assess if full recombinants (2.7 kb linear) or HJs were the predominant form produced (slowly migrating due to increased size and the χ-like shape). This analysis revealed that the predominant form of the apparent dimer was the HJ form, though about 30% was present as full recombinant product (**Fig 4**). The ability to produce some full recombinants would explain the ability of the hyperactive mutant recombinases to produce a ladder of multimer forms, since a reaction that could only progress to the HJ intermediate would be unable to produce any products larger than a dimer (see **Fig 2**). The χ-shaped product was confirmed to be a HJ by resolution with the HJ resolving enzyme T7-endonuclease I ([17]; **Fig 4B**). T7 endonuclease I is a junction resolving enzyme but can also digest DNAs with mismatches and act as a non-specific nuclease under conditions that promote overdigestion (higher reaction temperature and excess enzyme; see [18] and the NEB product page for the enzyme). The putative HJ was completely converted to products but there is evidence of some overdigestion as evidenced by the production of some smaller products than the expected 2.7 kb linear DNA. Perhaps some HJs were cleaved on more than one pair of strands or non-specific post-resolution reaction occurred (see Figs 5 and 6).

Since the hyperactive recombinase variants did not have the competing telomere resolution reaction operative like the TelA (D398A) mutant did we were able to examine recombination reactions of these mutants without gel purification (**Fig 5**). For the hyperactive recombinases it became apparent that they readily produce multimer products. Upon digestion with SspI alternate putative HJ forms with quite different gel mobilities became apparent (HJ_dim_ and HJ_mult_). Treatment of the SspI-linearized HJs with T7 endonuclease I resulted in the resolution of the HJs. We inferred that the two forms of the HJ derived from strand exchange between monomer plasmids to produce the HJ_dim_ form similar to that seen in **Fig 4**. The HJ_mult_ form likely derived from strand exchange of a recombinant dimer with monomer substrates or recombinant dimers producing a higher molecular weight product with multiple HJs (**Fig 5**). In support of this contention is the absence of HJ_mult_ in the reaction of TelA (D398A) that produced no recombinant multimers had only the HJ_dim_ form (**Fig 4**). We also ran a timecourse of a reaction with the (ΔN; D202RE337KD398A) mutant and found the incidence of the HJ_mult_ form correlates with the production of multimeric recombinant forms (**S6 Fig** in S1 File). Within each HJ we also expect there will be forms resulting from synapsis of the recombining partners in direct *vs.* inverted repeat orientation (**Fig 5A**). We expect this since tyrosine recombinases require reactions sites with some asymmetry between the scissile phosphates to provide minimal directionality cues for recombination; our replicated telomere (*rTel*) junctions, instead, possess perfect inverted repeat symmetry [9,19,20]. The products of resolution were, unexpectedly, 2.1 and 0.6 kb in size rather than 2.7 kb indicative of resolution of the HJs by cleavage of all four strands rather than the usual resolution via cleavage of two strands only (**Fig 5B**).

We gel-purified the two HJ forms (HJ_dim_ and HJ_mult_) as well as a much more slowly migrating band designated HJ* that were apparent in concentrated recombination reactions promoted by the (ΔC3; D202RE337KD398A) mutant reacting with negatively supercoiled wild type plasmid (pEKK494). We then subjected these gel-purified forms to analysis by treatment with the HJ resolving enzyme T7 endonuclease I. This analysis performed without the background of multiple bands allowed us to more confidently assign the products to resolution of the individual HJ forms. HJ_dim_ and HJ_mult_ both resolve primarily into products sized 2.1 kb and 0.6 kb in size, indicative of action by T7 endonuclease I to cleave all four strands instead of the more standard resolution products expected of linear monomer sized 2.7 kb that would be obtained by cleavage of a pair of strands (**Fig 6**). This non-standard resolution may result from the unusual symmetry of the resulting HJs. The more complex higher molecular weight HJ* resolves more cleanly into products expected from two strand resolution of HJs formed by snyapsis in the two possible orientations; 2.7 kb for antiparallel synapsis and 4.2/1.2 kb products from synapsis in a parallel orientation (**Fig 6A**). However, resolution of HJ* also produces a majority of its products by cleavage of 4 strands (2.1/0.6 kb products; **Fig 6**).

### Asymmetrizing the Agrobacterial *rTel* junction does not produce directional strand exchange

Previous study of *Borrelia* ResT indicated that wild type ResT could promote HJ formation between 2 *rTel*s present in the same supercoiled plasmid substrate [9]. Substrates with *bona fide rTel*s with complete inverted repeat symmetry produced HJs that, when resolved, produced both inversion and deletion products. Introducing asymmetry between the scissile phosphates to turn the *rTel*s into sites that mimic the directionality cues present in tyrosine recombinase substrate sites (like Cre’s *loxP* sites) resulted in imposition of reaction directionality to the resulting recombination reactions, while simultaneously inhibiting telomere resolution [9].

**Fig 7:**
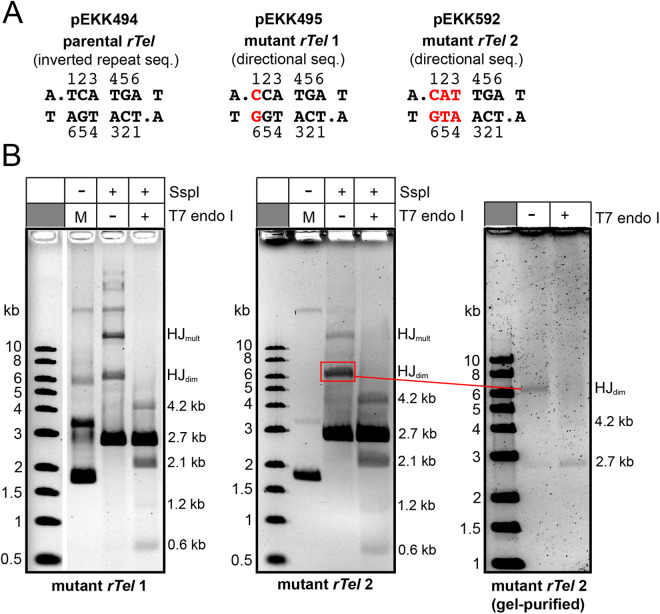
Analysis of HJ’s produced with mutant *rTels* possessing sequence asymmetry between the scissile phosphates. A) Schema of the sequence between the scissile phosphates for the parental *rTel* sequence that possesses inverted repeat symmetry *vs* two mutant *rTel*s that disrupt the inverted repeat symmetry of the parent via mutations at position 1 (mutant *rTel* 1; pEKK495) and at positions 1, 2- & 3 (mutant *rTel* 2; pEKK592). B) 0.8% agarose 1X TAE gel panels of the reaction of the (ΔN; D202RE337KD398A) mutant with mutant *rTel* 1(pEKK495) and mutant *rTel* 2 (pEKK592) using 30^o^C, 2 h incubations followed by digestion of the reactions with SspI and by treatment at 30^o^C, 2 min with T7 endonuclease I. M denotes a mock incubation of supercoiled pEKK495 or pEKK592 without TelA addition or subsequent treatment; HJ denotes Holliday junctions (forms noted in the legend of **Fig 6**); the numbers to the right of the gel indicate the size of the products noted in kb. The ethidium bromide and SyberGold stained gels are shown as a inverted images. The rightmost gel panel shows T7 endonuclease I treatment of gel-purified SspI-digested HJ produced by reaction with pEKK592 (mutant *rTel* 2); this gel panel was stained with SyberGold.

We tried to provide a similar modest directionality cue in the substrate by introducing a single point mutation between the scissile phosphates by changing the wild type sequence from TCATGA to CCATGA (pEKK495; mutant *rTel* 1). This change produces an *rTel* that cannot form hairpin telomeres due to the asymmetry introduced in the sequence in a position what would have to form the first basepair in the developing hairpins during telomere resolution. We found that reactions with mutant *rTel* 1 with a hyperactive recombinase variant of TelA produced essentially the same products as reactions with wild type substrate but produced them in lower yield (**Fig 7B; left panel**).

Since asymmetrizing the sequence between the scissile phosphates at only the first nucleotide failed to impose directionality upon the strand exchange reaction promoted by the TelA mutants we wanted to test a substrate with a more radical asymmetrization that disrupted the symmetry at the first, second and third nucleotides (CCTTGA mutant sequence instead of the TCATGA wild type; mutant *rTel* 2 present in pEKK592).

Initial characterization of mutant *rTel* 2 indicated that despite the directionality cue provided by the *rTel* TelA mutants with a recombinase phenotype were still not restricting synapsis and strand exchange between *rTel*s synapsed in an antiparallel orientation (**Fig 7B; middle panel**). This is clear from the robust yield of HJs that resolve into 4.2/1.2 kb products when treated with T7 endonuclease I. These are products expected for HJs produced by strand exchange between pEKK592s brought together in a parallel synapse. Because of the large amount of unreacted substrate remaining after the reaction it was unclear if the HJ was also resolved into the 2.7 kb products expected from HJs formed by synapsis and strand exchange in the antiparallel orientation that is the norm for tyrosine recombinases. To remedy this issue we managed to gel purify a small amount of the HJ generated with mutant *rTel* 2 and treated it with T7 endonuclease I (**Fig 7B; right panel**). We were able to visualize resolution to the 2.7 kb products expected from HJs formed by synapsis and strand exchange in the antiparallel orientation. The lack of background cleavage by T7 endonuclease I of SspI-linearized mutant *rTel* 1 and mutant *rTel* 2 strengthens the interpretation that the products seen after T7 endonuclease I treatment of reactions with these plasmids are derived from resolution of the resulting HJs (**S7 Fig** in S1 File).

### The ResT (D328A) mutation suppresses telomere resolution but allows recombination at replicated telomeres (*rTels*)

We have previously reported mutation of the equivalent aspartic acid residue in ResT (D328A) assayed with oligonucleotide *rTel* substrates [16]. The D328A mutant was found to be inactive for telomere resolution. This mutant and others were readily rescued by *rTel* modifications that mimicked underwinding between the scissile phosphates (missing bases or mismatches). We interpreted this as the involvement of this constellation of ResT residues in forming an underwound pre-cleavage intermediate that allows DNA cleavage followed by strand ejection to promote the forward reaction in preference to just resealing the DNA in a topoisomerase IB-like activity. Most of the mutants were cleavage defective on unmodified substrate. However, ResT (D328A) was found to be cleavage competent. Its modeled position close to the scissile phosphates prompted us to hypothesize that the D328 residue to be key for strand ejection as well. TelA possesses a highly similar constellation of residues with broadly shared phenotypes when mutated [14].

**Fig 8:**
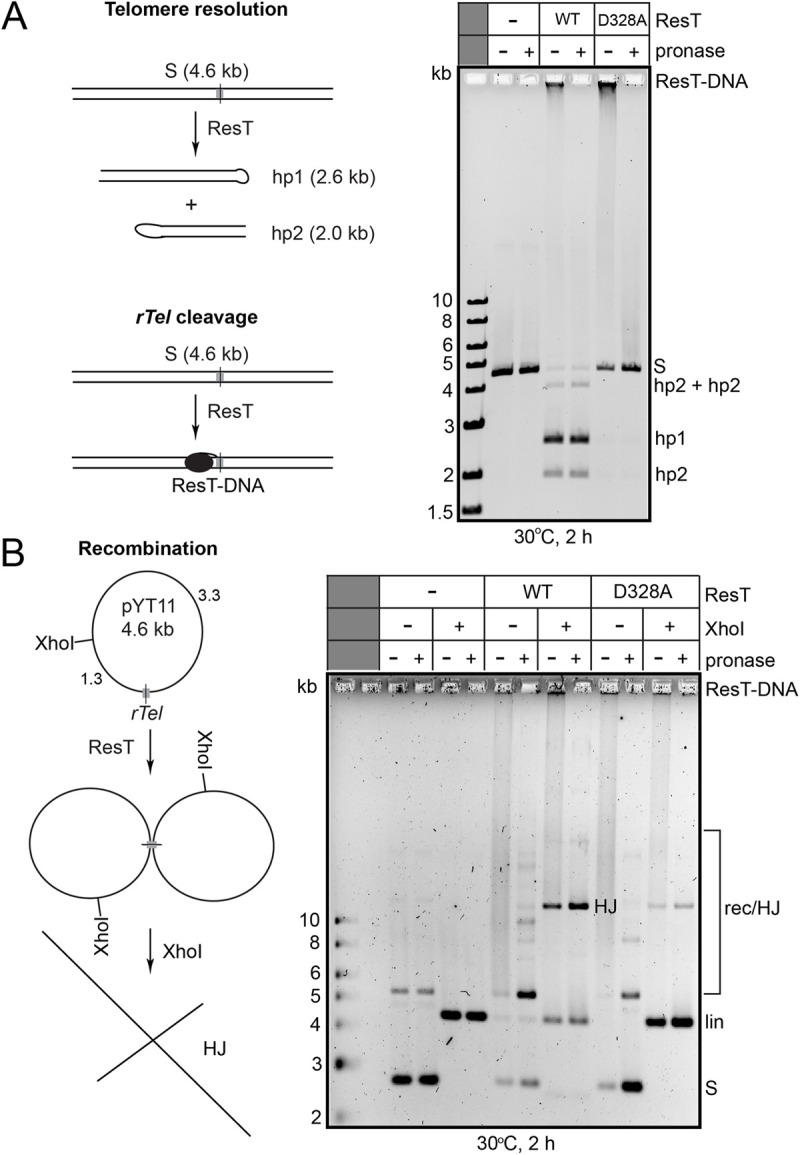
ResT (D328A) is inactive for telomere resolution but retains some ability to promote recombination. A) Left: schematics of a telomere resolution reaction and a reaction wherein ResT has cleaved only one strand of the substrate *rTel*. Right: presented is a 0.7% agarose 1X TAE gel analysis of 30^o^C 2 h incubations of 150 nM of wild type or ResT (D328A) with XhoI-linearized pYT11 substrate plasmid (2 μg/mL). ResT-DNA denotes probable substrate plasmid with ResT covalently attached after cleavage of one strand; S denotes the XhoI-linearized substrate plasmid; hp1 and hp2 denote the products of telomere resolution; hp2 + hp2 denotes the post-resolution fusion of hp2 products [22]. B) Left: schematic of recombination with supercoiled pYT11 to produce a HJ that is visualized by digestion with XhoI after treatment with ResT. Right: presented is a 0.7% agarose 1X TAE gel analysis of reaction of 150 nM of wild type or ResT (D328A) reacted with supercoiled pYT11 substrate plasmid (2 μg/mL). Shown are the results from 30^o^C, 2 h incubations. S denotes supercoiled plasmid substrate; lin denotes XhoI-linearized substrate; rec/HJ denotes the HJ formed by strand exchange between plasmid copies; HJ denotes the XhoI-digested form of the HJ.

We assayed the telomere resolution and recombination activity of ResT (D328A) on plasmid substrates to test if this mutation also promoted a switch from telomere resolution activity to recombination in the ResT system (**Fig 8**). For telomere resolution assays we employed a substrate plasmid with a single *rTel* junction present (pYT11) linearized with the single-hit enzyme XhoI (**Fig 8A**; [21]). For recombination assays we used a negatively supercoiled version of the same plasmid (**Fig 8B**).

Telomere resolution reactions with wild type ResT produced the expected hp telomere products while reaction with ResT (D328A) produced a prominent well-shift that returned to unreacted substrate when the reaction was treated with the protease mixture, pronase, prior to loading (**Fig 8A**). These results are consistent with previous results that indicated that ResT (D328A) was capable of cleaving the *rTel* but not of resolving it [16]. However, the well-shift and migration position of the pronase digested reaction is most consistent with a scenario where the enzyme has cleaved only one strand of the *rTel* and then rather than resealing the cleaved strand has remained covalently attached to the *rTel* rather than resealing the strand to reconstitute the substrate DNA. Cleaving both strands but failing to complete resolution would release double-strand break products after pronase digestion if both strands had been cleaved. This is unexpected behaviour since DNA cleavage and strand exchange by telomere resolvases are normally concerted events [8]. Cleavage of only a single strand of an *rTel* may help explain why this mutation was found to be permissive for recombination but not telomere resolution since recombination is characterized by cleavage and strand exchange of one strand at a time in the recombining DNA partners while telomere resolution requires concerted cleavage and strand exchange of both strands in an *rTel* that is being resolved (**Fig 8B**).

Recombination reactions with supercoiled pYT11 yielded HJs with both wild type ResT and ResT (D328A), though the yield with wild type ResT was much higher. Wild type ResT, but not the D328A mutant, also showed a small amount of competing telomere resolution occurring as well (**Fig 8B**). So, while the D328A mutation in ResT has abolished telomere resolution it still permitted a small amount of site-specific recombination between *rTel*s to occur leading to a HJ intermediate. In order to obtain a more active recombinase version of ResT the D328A mutation will likely need to be combined with mutations that raise the general activity of the enzyme.

## Conclusions

Telomere resolvases employ the same reaction chemistry for cleaving and rejoining DNA strands as that employed by type-IB topoisomerases and tyrosine recombinases. The differences between the products produced by these three families of enzymes is largely a function of difference in the number of strands cleaved and the identity of the strands they are rejoined to; this is tied to the oligomeric state of the enzymes when they are performing their reactions [1]. Telomere resolution requires dimerization on a replicated telomere junction to produce the pair of hp telomere products. However, the telomere resolvases from *Borrelia burgdoferi* (ResT) and *Agrobacterium tumefaciens* (TelA) have both been observed to act as site-specific topoisomerases or site-specific recombinases under certain conditions, implying a degree of flexibility in oligomerization and coordination between the DNA cleavage and rejoining events [8,9,11,12].

For ResT, the recombinase activity is readily observed with wild type enzyme. The key determinant of whether telomere resolution or recombination occurs appears to be the presence and sign of DNA supercoiling in the substrate DNA [9,10]. The presence of negative supercoiling suppresses telomere resolution and promotes recombination [3,9]. The presence of positive supercoiling suppresses recombination and, instead, strongly stimulates telomere resolution [9,10]. For TelA the recombinase activity is cryptic, requiring mutation of the enzyme to overcome autoinhibition that suppresses the competing reactions of reaction reversal (fusion of hp telomeres) and recombination between *rTel*s [11,12]. Even with mutations that overcome autoinhibition a very sensitive assay is required to detect recombination by these mutants [12].

In the present study we report the discovery of a conserved catalytic domain aspartic acid residue that makes contacts with the substrate DNA near the scissile phosphates in both TelA and ResT. Mutation of this aspartic acid in both TelA and ResT results in suppression of telomere resolution while allowing recombination. For TelA, the D398A mutation is sufficient, in combination with the presence of negative supercoiling in the substrate DNA, to promote recombination between *rTel*s without mutations that have been reported to overcome autoinhibition (**Figs 2**–**4**). Combination of the D398A mutation with hyperactivating mutations known to overcome autoinhibition, unexpectedly, gave rise to TelA variants that are inactive as telomere resolvases but are instead hyperactive recombinases (**Figs 5–7**). Mutation of the corresponding D328 residue to alanine in ResT abolished telomere resolution but still allowed some recombination to occur, likely due to the D328A mutation’s propensity to support DNA cleavage of only a single strand in an *rTel* (**Fig 8A**).

While the discovery of reaction conditions and enzyme mutations that switch a telomere resolvase to a recombinase highlights the intrinsic flexibility of the telomere resolvases and hints at the possible origin of these enzymes as mutated tyrosine recombinases, the recombination they promote is too aberrant to make them useful as biotechnology tools for the controlled production of hairpins or recombinants. The reactions accumulate the HJ intermediate of recombination rather than the more useful full recombinants (**Figs 4–7**). In the case of TelA where some full recombinants are recovered both the products of strand exchange from parallel and antiparallel synapses were produced. This was the case even when the *rTel*s were asymmetrized between the scissile phosphates to make them more closely resemble the reaction sites of a simple recombinase like Cre (**Fig 7**). Additionally, even when full recombinant dimers are produced by the hyperactive TelA recombinase variants they tended to participate in further rounds of recombination to produce higher-order multimers rather than reaching a more useful equilibrium between dimers and monomers as is typical of recombinases like Flp or Cre (**Figs 5–7**).

Previous studies with ResT successfully imposed reaction directionality (to produce inversions or deletions) when the *rTel*s were asymmetrized by mutation of the sequence between the scissile phosphates to disrupt the perfect inverted repeat symmetry of the *rTel*s [9]. This study’s discovery that the D328A mutation in ResT suppresses telomere resolution while allowing recombination points to combination of this mutation with hyperactivating mutations as the most promising avenue to pursue to produce a biotechnology tool based on a single enzyme that can either produce hp telomeres or recombinants on minor variations of the same target sequence.

## Supporting information

S1 Raw Images (PDF)

S1 File Supporting information. (PDF)
