# Supplementary material for: Conversion of a telomere resolvase into a Cre-like site-specific recombinase
Source: PLoS One. 2025 Jul 17;20(7):e0328478. doi: 10.1371/journal.pone.0328478 (PMC12270096; doi:10.1371/journal.pone.0328478)
Supplement: S1 Raw Images — (PDF) [file pone.0328478.s001.pdf]

S1 raw images

Fig 2 raw images

A

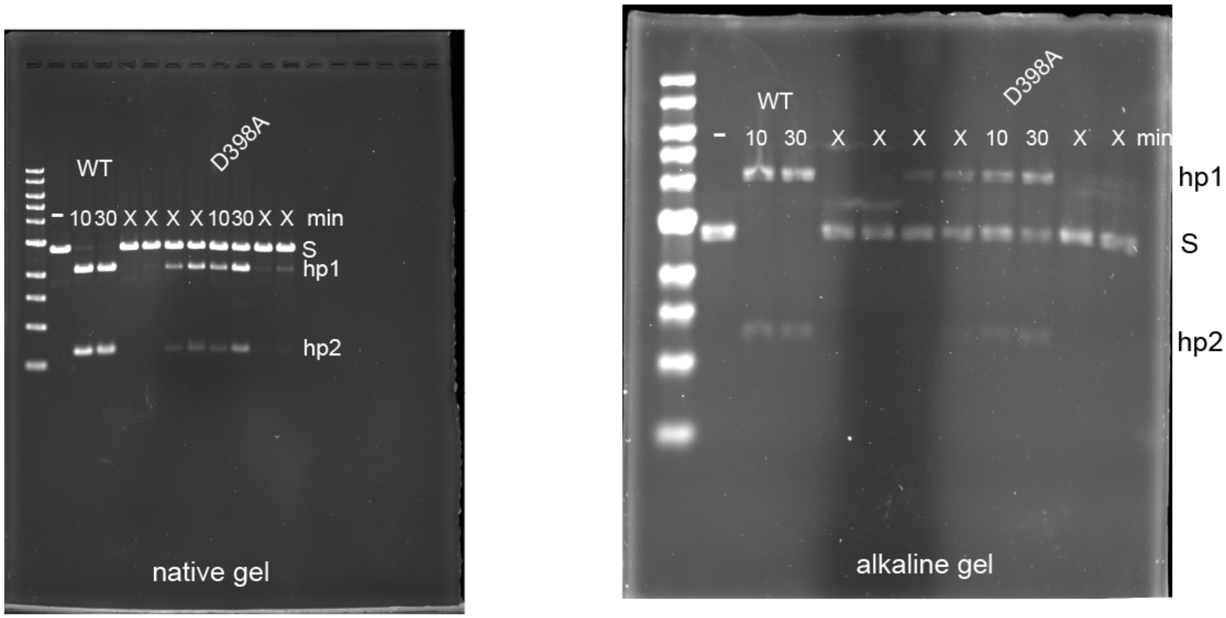

B

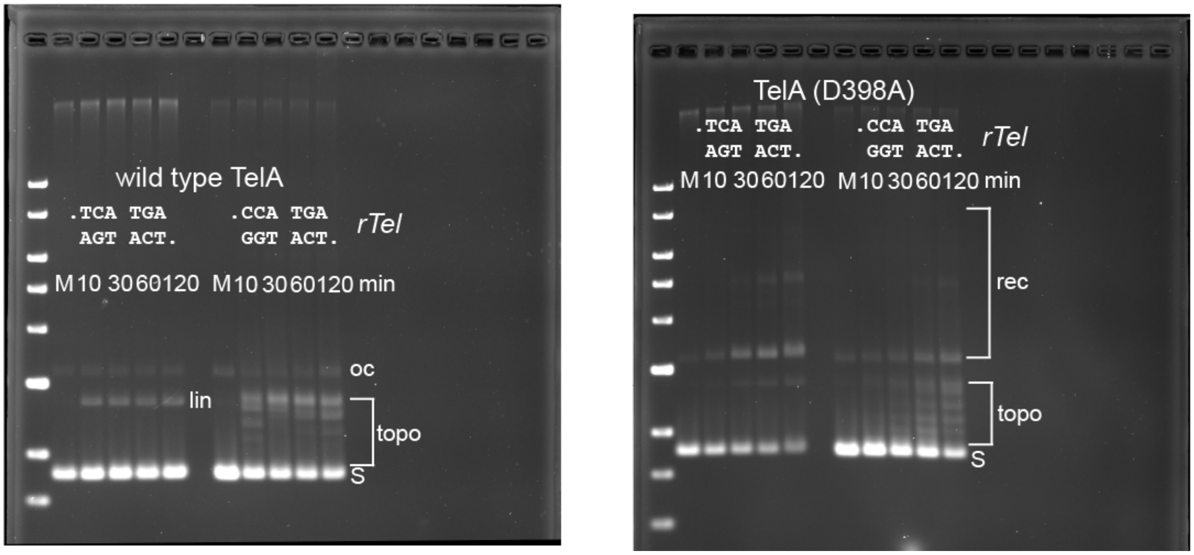

Uncropped gels for Fig 2. Where they appear X’s mark lanes of mutants not reported in this study.

Fig 3 raw images

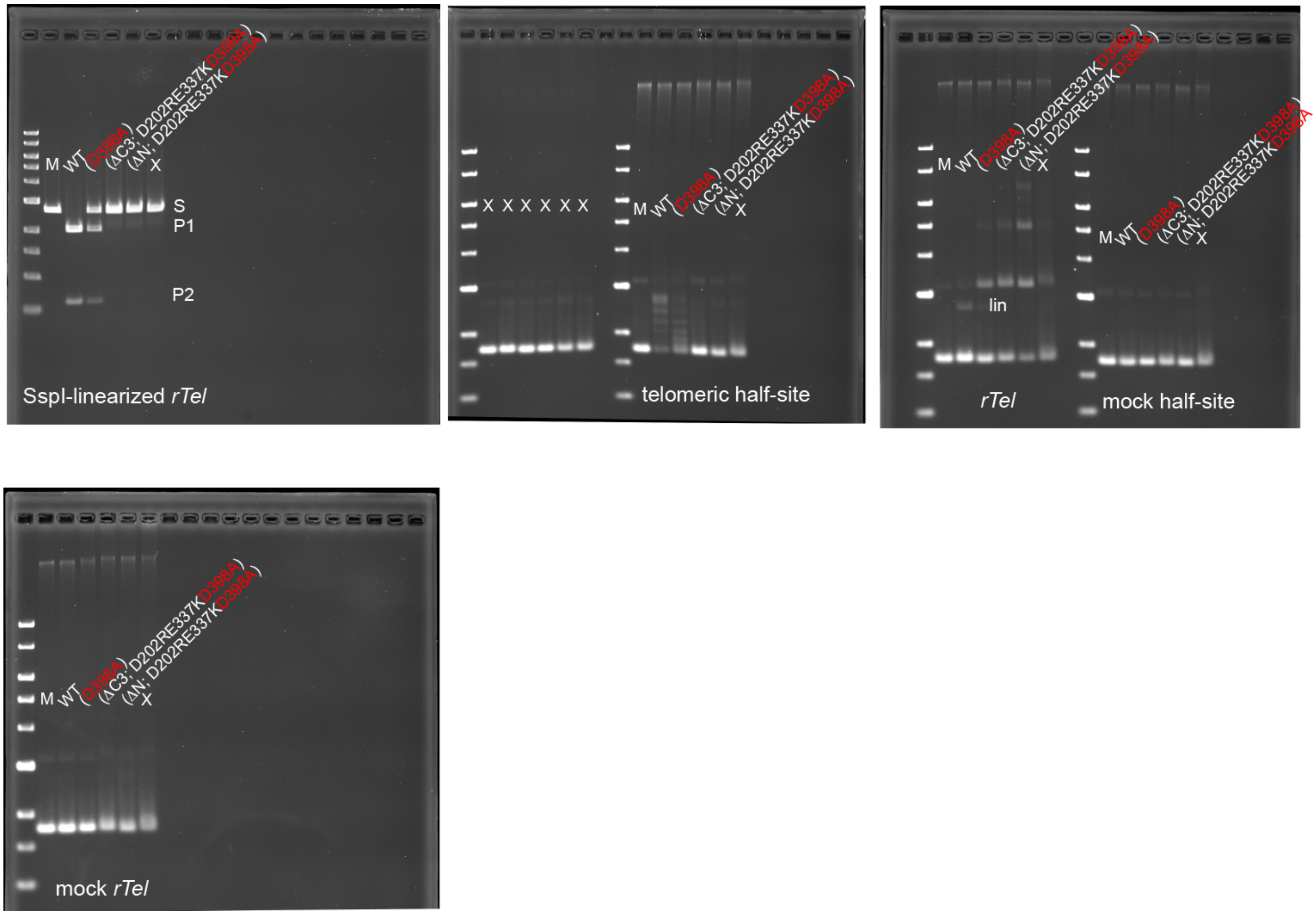

Uncropped gels for Fig 3. Where they appear X's mark lanes of mutants not reported in this study.

Fig 4 raw images

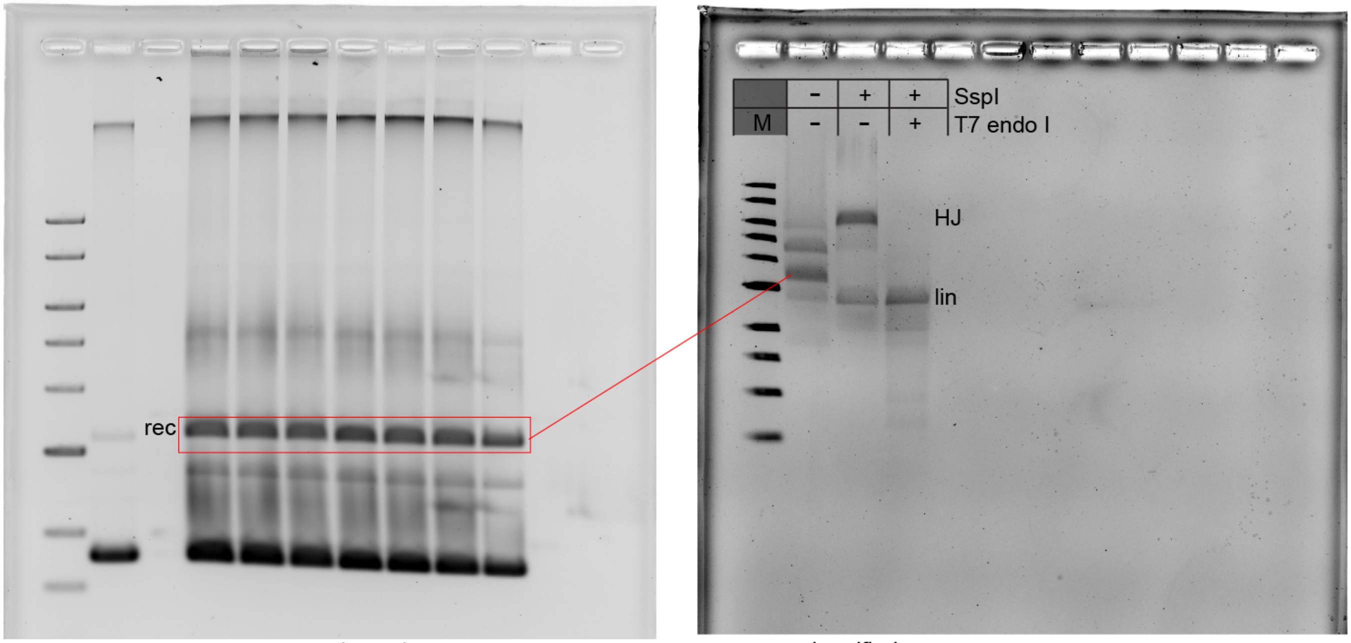

preparative gel

gel purified rec

Uncropped gels for Fig 4.

Fig 5 raw images

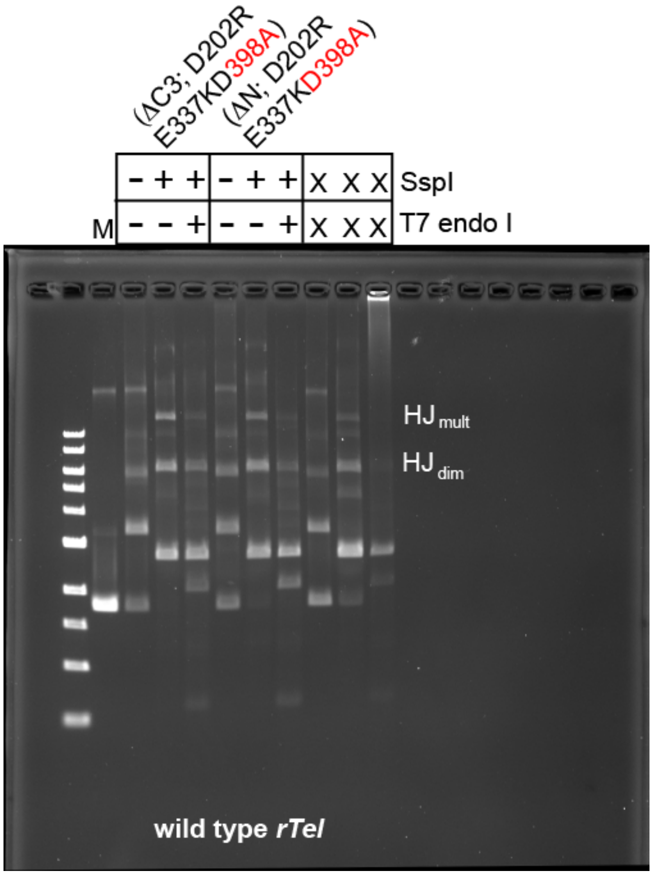

Uncropped gel for Fig 5. Where they appear X's mark lanes of mutants not reported in this study.

Fig 6 raw images

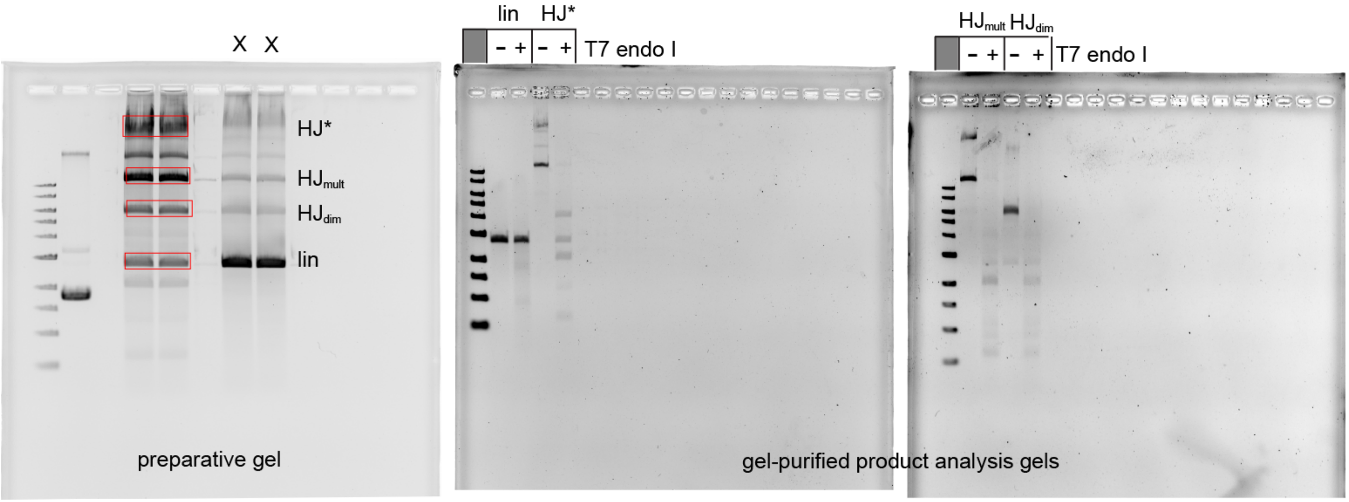

Uncropped gels for Fig 6. Where they appear X's mark lanes of mutants not reported in this study.

Fig 7 raw images

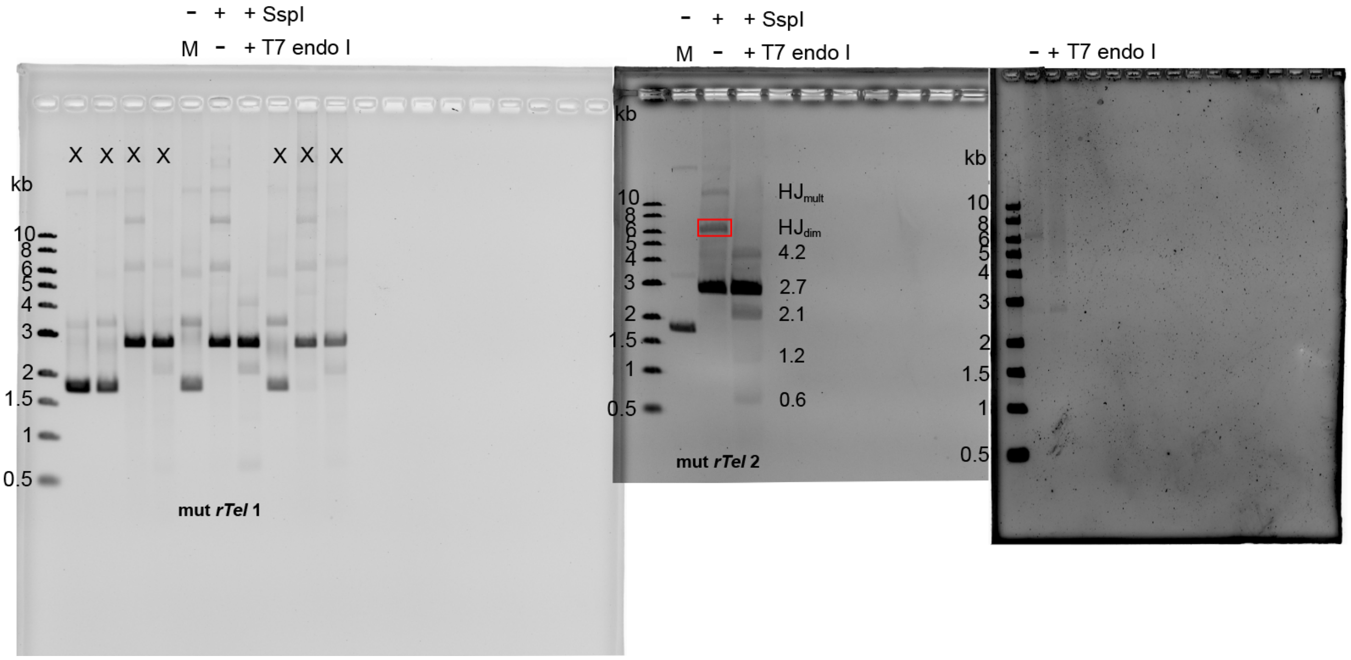

Uncropped gels for Fig 7. Where they appear X's mark lanes of mutants not reported in this study.

Fig 8 raw images

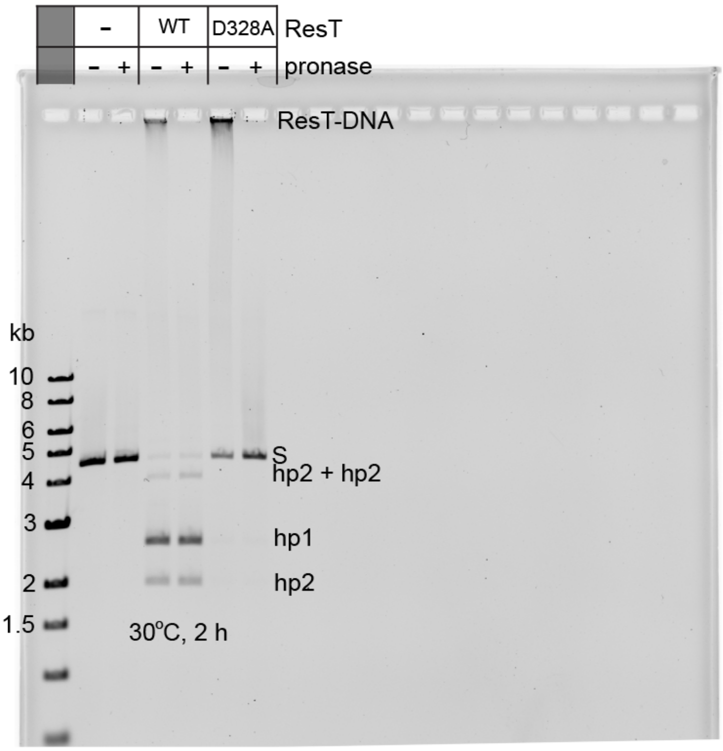

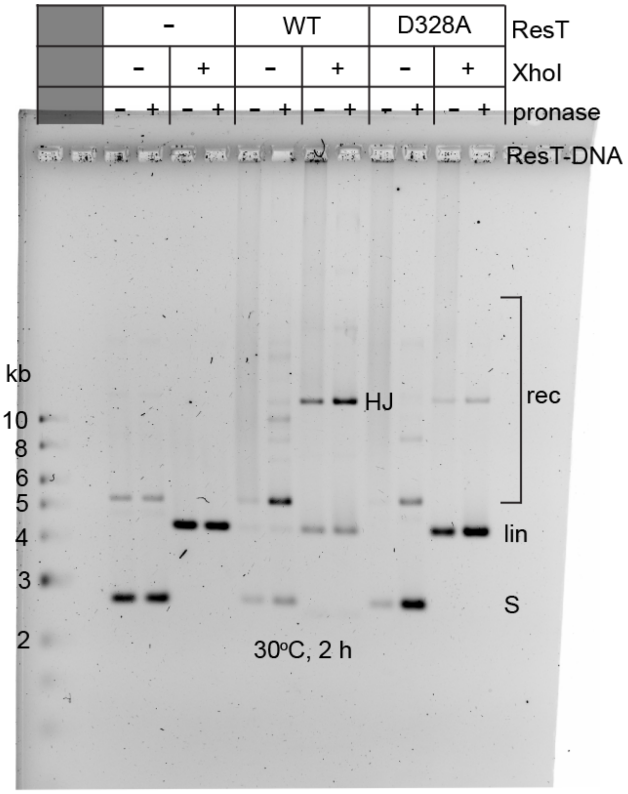

Uncropped gels for Fig 8.

S2 Fig raw images

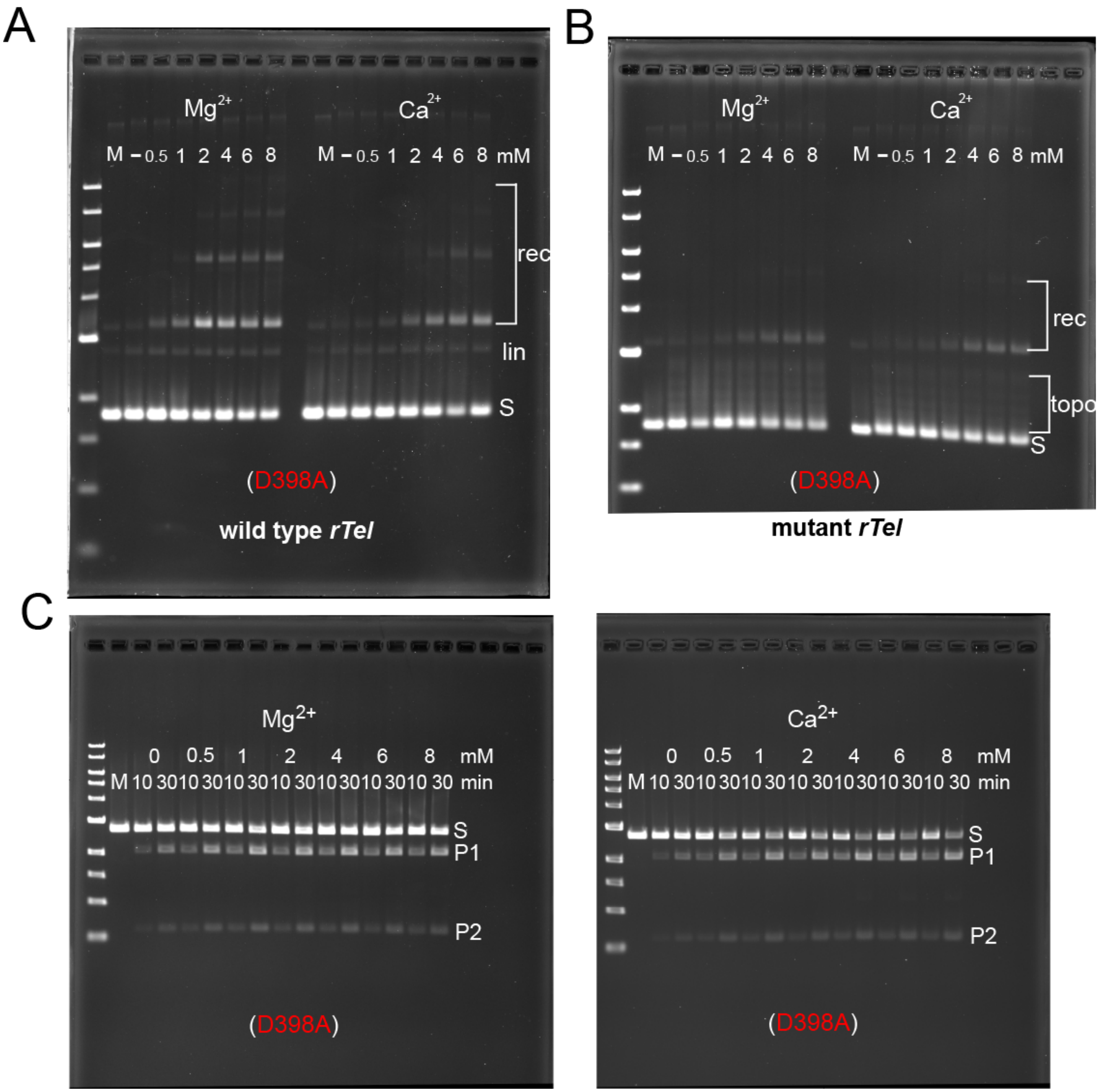

Uncropped gels for S2 Fig.

S3 Fig raw images

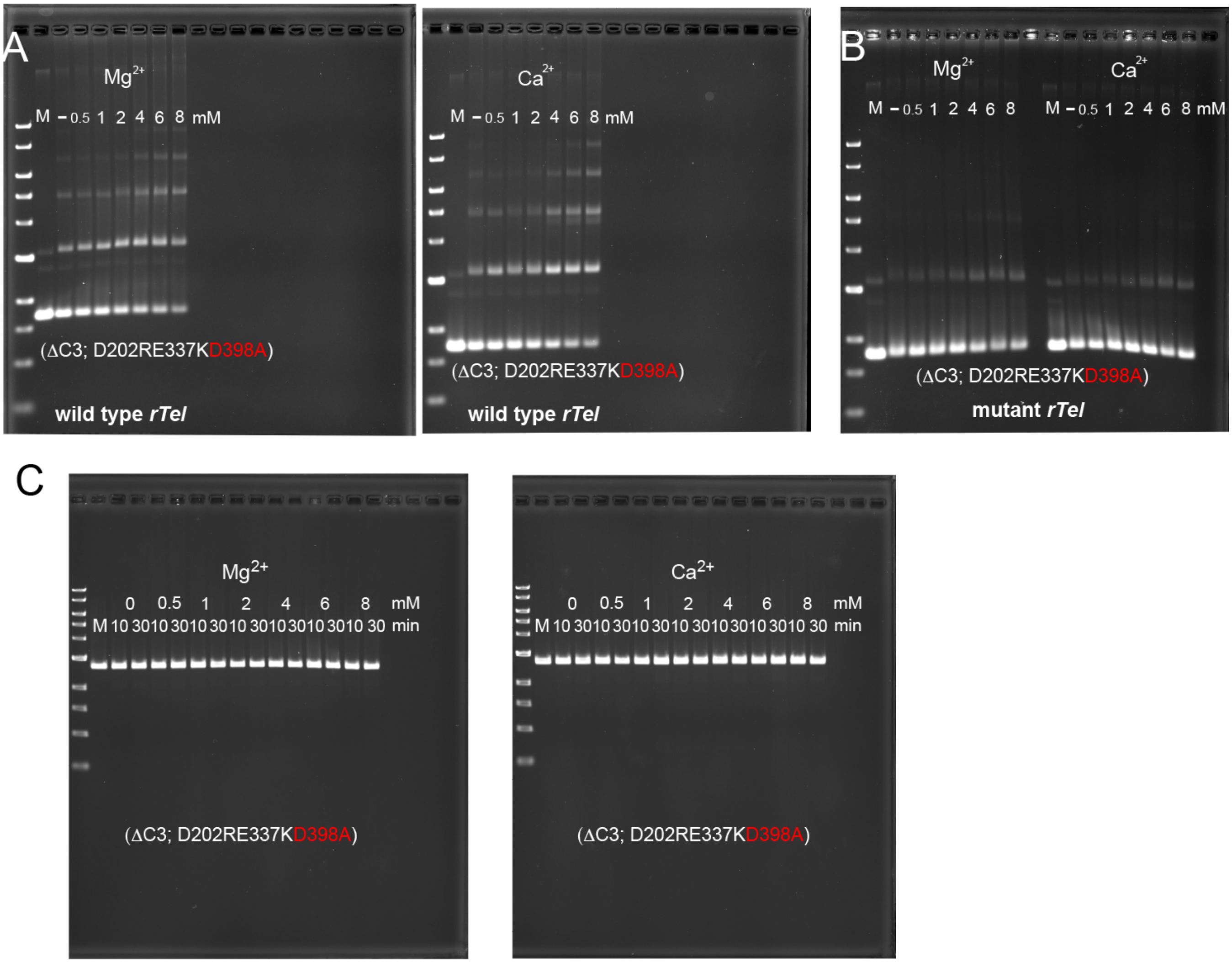

Uncropped gels for S3 Fig.

S4 Fig raw images

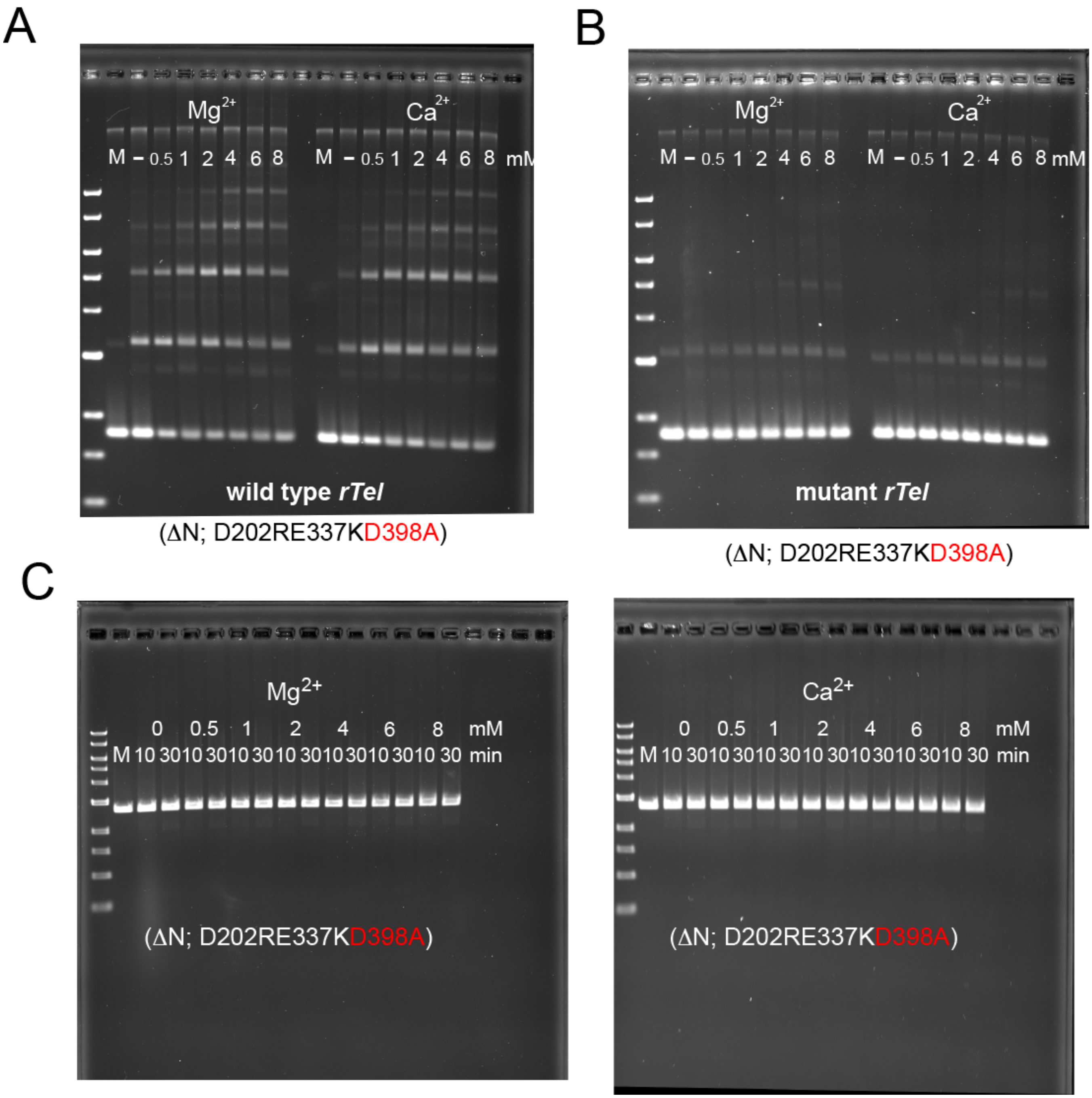

Uncropped gels for S4 Fig.

S6 Fig raw images

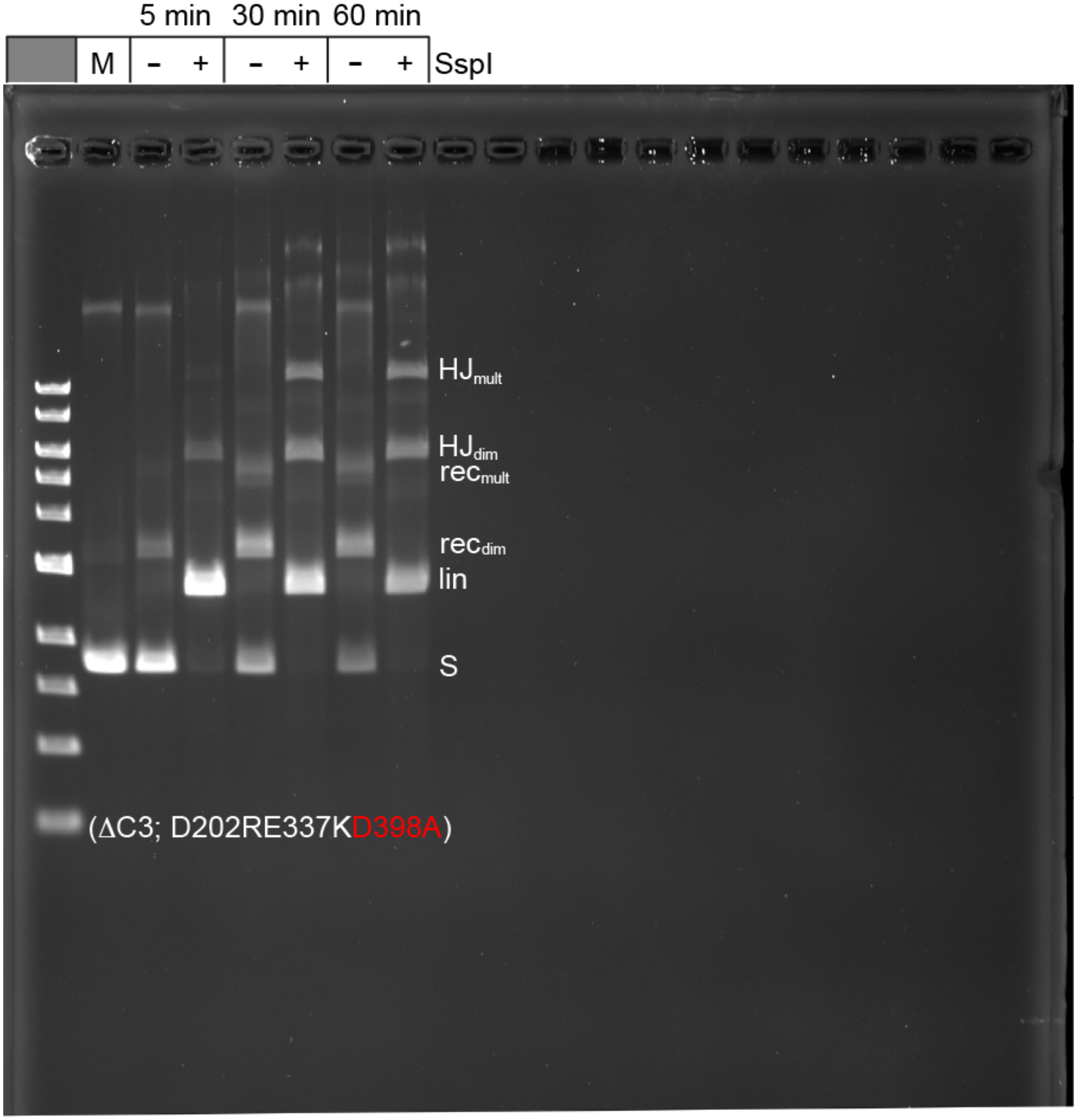

Uncropped gel for S6 Fig.

S7 Fig raw images

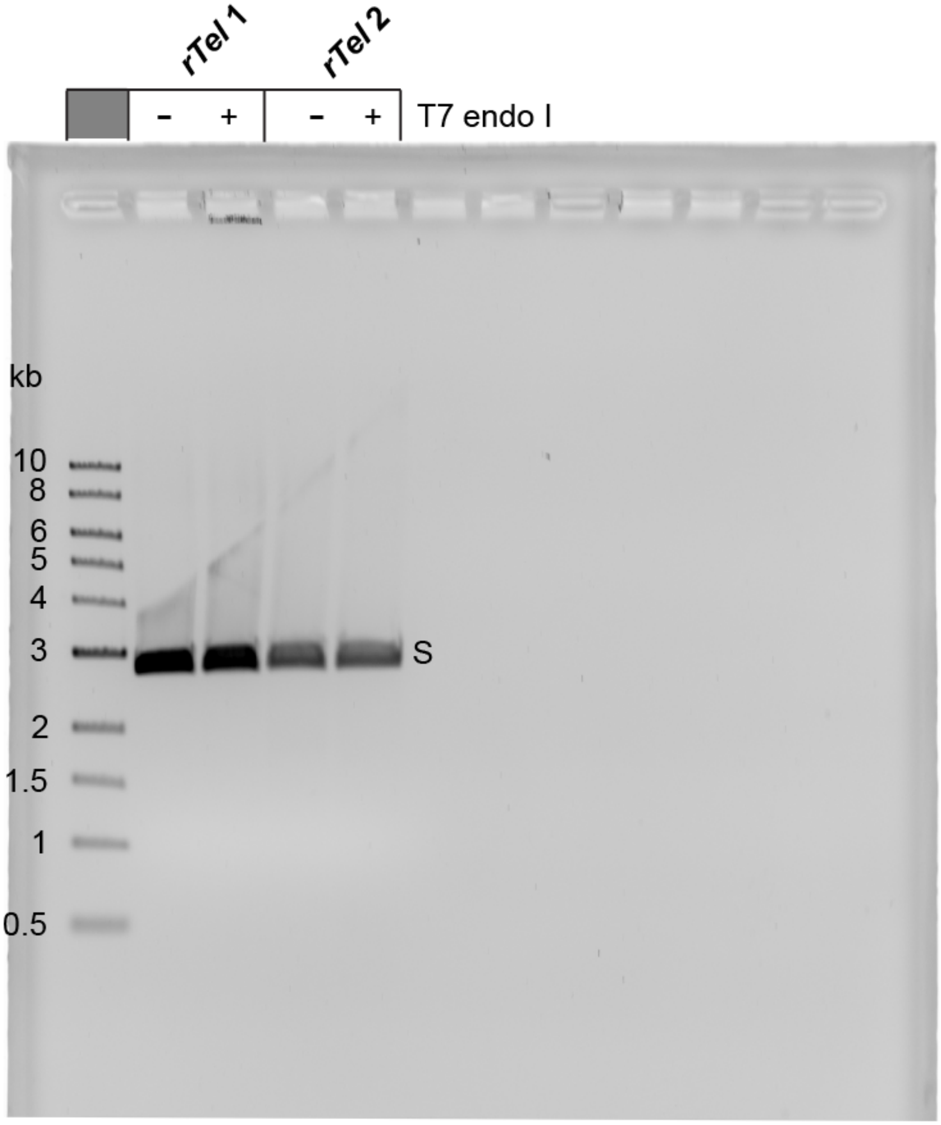

Uncropped gel for S7 Fig.
