# Supplementary material for: Conversion of a telomere resolvase into a Cre-like site-specific recombinase
Source: PLoS One. 2025 Jul 17;20(7):e0328478. doi: 10.1371/journal.pone.0328478 (PMC12270096; doi:10.1371/journal.pone.0328478)
Supplement: S1 File — (PDF) [file pone.0328478.s002.pdf]

Supporting information

A

TelA domain structure

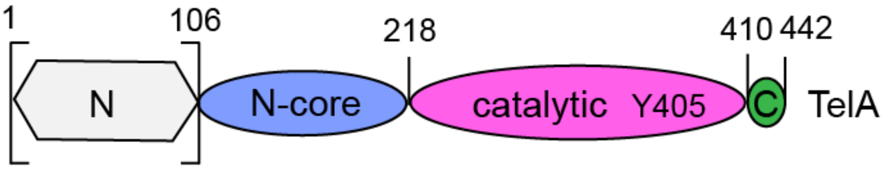

B

Recombination activating mutation D398A location

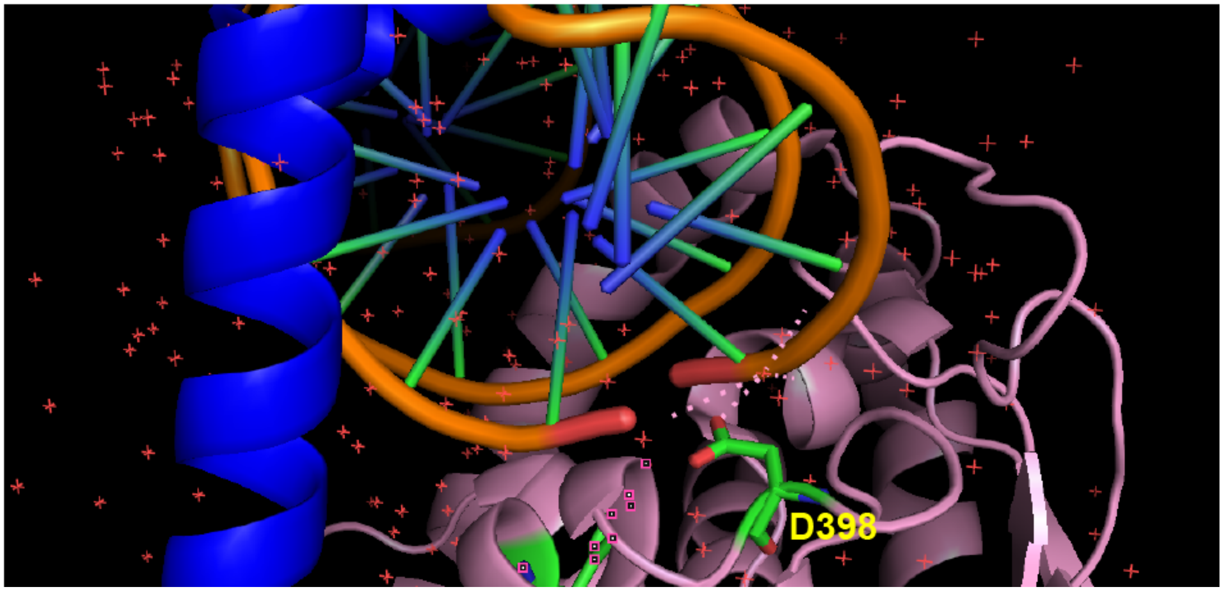

S1 Fig. TelA's D398 residue makes water-mediated contacts with the DNA backbone near the scissile phosphate.

A) Schematic of the domain organization of TelA. The D398 residue resides in the catalytic domain (shaded pink) that is shared by all telomere resolvases and that is related to the catalytic domain to type IB topoisomerases and tyrosine recombinases.

B) Shown is a close-up view of D398's water-mediated interactions with the backbone of the DNA near the scissile phosphate. The model is derived from a co-crystal structure of TelA with hairpin telomere DNA. The scissile phosphate is shaded in red, the red crosses represent water molecules and the dashed lines the polar contacts. The TelA catalytic domain is coded as pink and the hairpin-binding module of the N-core domain is the helix coded in blue. The model was built with PyMol using pdb accession# 4e0g.

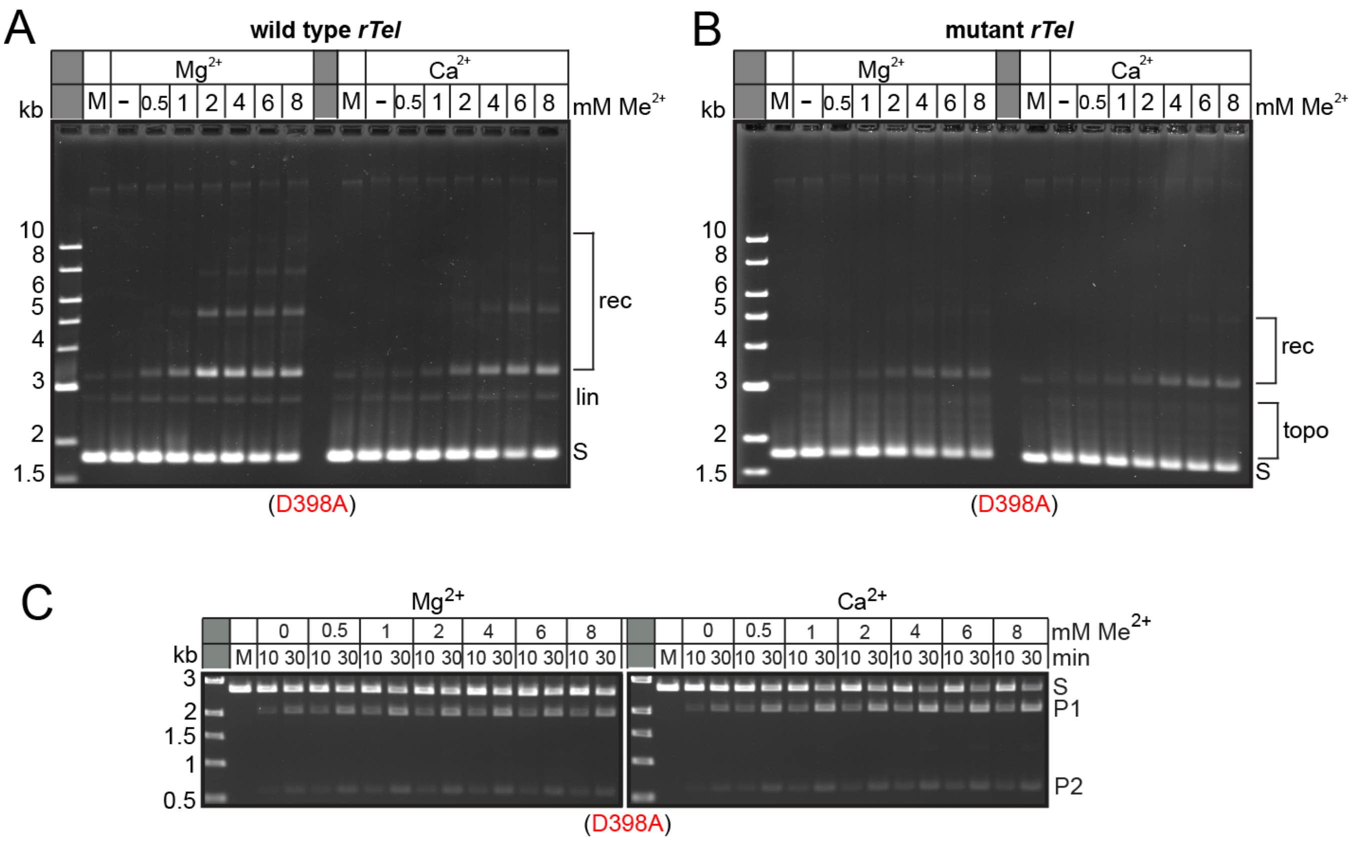

S2 Fig. The D398A mutation activates TelA as a recombinase.

A) 0.8% agarose 1X TAE gel panels of divalent metal ion titrations of the TelA (D398A) mutant reacted with negatively supercoiled plasmid substrate (wild type; pEKK494) incubated at 30°C for 30 min.

B) 0.8% agarose 1X TAE gel panel of divalent metal ion titrations of the TelA (D398A) mutant reacted with negatively supercoiled mutant plasmid substrate (pEKK495) incubated at 30°C for 30 min. S denotes substrate; rec denotes multimers of recombined plasmids; lin denotes the position that the linear product of telomere resolution; topo denotes a ladder of topoisomers.

C) 0.8% agarose 1X TAE gel panels of divalent metal ion titrations of the TelA (D398A) mutant reacted in telomere resolution reactions with SspI-linearized wildtype plasmid substrate (pEKK494) at 30°C for 10 min. S denotes substrate; P1 & P2 denote the migration position of the expected products of telomere resolution.

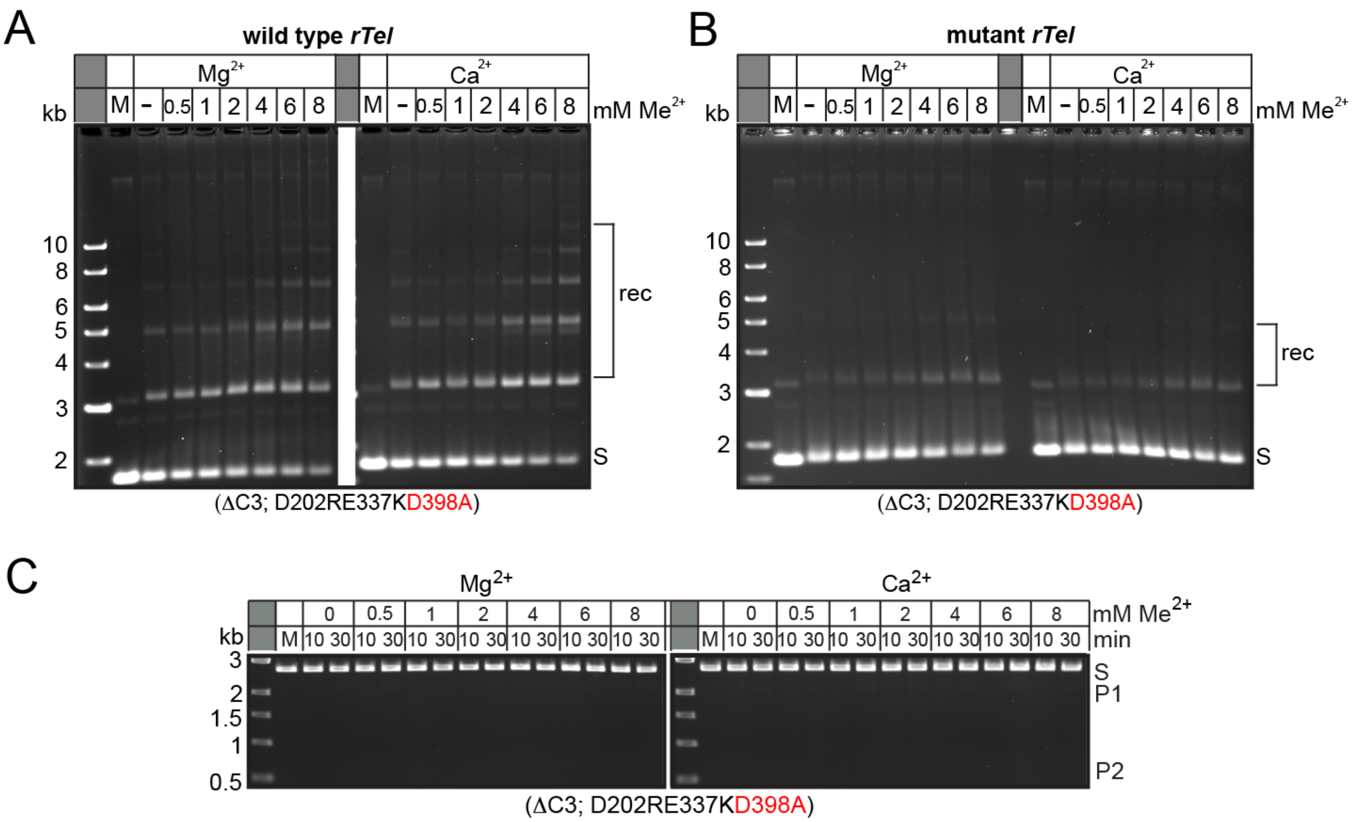

S3 Fig. Combining the D398A mutation with mutations that hyperactivate TelA for telomere resolution activates TelA as a recombinase.

A) 0.8% agarose 1X TAE gel panels of divalent metal ion titrations of the (ΔC3; D202RE337KD398A) mutant reacted with negatively supercoiled plasmid substrate (wild type; pEKK494) incubated at 30°C for 10 min.

B) 0.8% agarose 1X TAE gel panel of divalent metal ion titrations of the (ΔC3; D202RE337KD398A) mutant reacted with negatively supercoiled mutant plasmid substrate (pEKK495) incubated at 30°C for 10 min. S denotes substrate; rec denotes multimers of recombined plasmids; lin denotes the position that the linear product of telomere resolution would migrate at.

C) 0.8% agarose 1X TAE gel panels of divalent metal ion titrations of the (ΔC3; D202RE337KD398A) mutant reacted in telomere resolution reactions with SspI-linearized wild type plasmid substrate (pEKK494) at 30°C for 10 min. S denotes substrate; P1 & P2 denote the migration position of the expected products of telomere resolution.

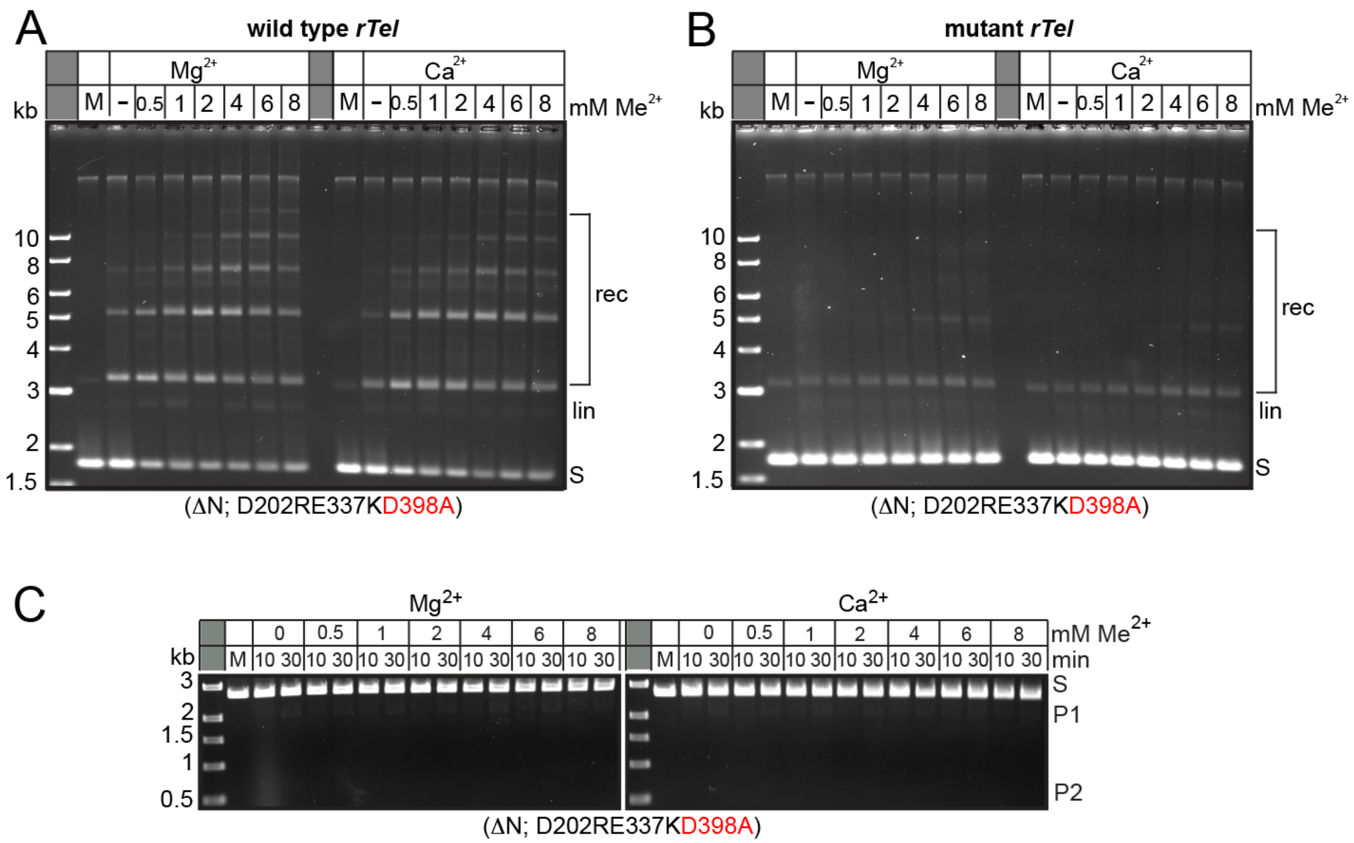

S4 Fig. Combining the N-terminal domain deletion with the D398A mutation switches TelA from a telomere resolvase to a recombinase.

A) 0.8% agarose 1X TAE gel panels of divalent metal ion titrations of the (ΔN; D202RE337K D398A) mutant reacted with negatively supercoiled plasmid substrate (wild type; pEKK494) incubated at 30°C for 10 min.

B) 0.8% agarose 1X TAE gel panel of divalent metal ion titrations of the (ΔN; D202RE337KD398A) mutant reacted with negatively supercoiled mutant plasmid substrate (pEKK495) incubated at 30°C for 10 min. S denotes substrate; rec denotes multimers of recombined plasmids; lin denotes the position that the linear product of telomere resolution would migrate at.

C) 0.8% agarose 1X TAE gel panels of divalent metal ion titrations of the (ΔN; D202RE337KD398A) mutant reacted in telomere resolution reactions with SspI-linearized wild type plasmid substrate (pEKK494) at 30°C for 10 min. S denotes substrate; P1 & P2 denote the migration position of the expected products of telomere resolution.

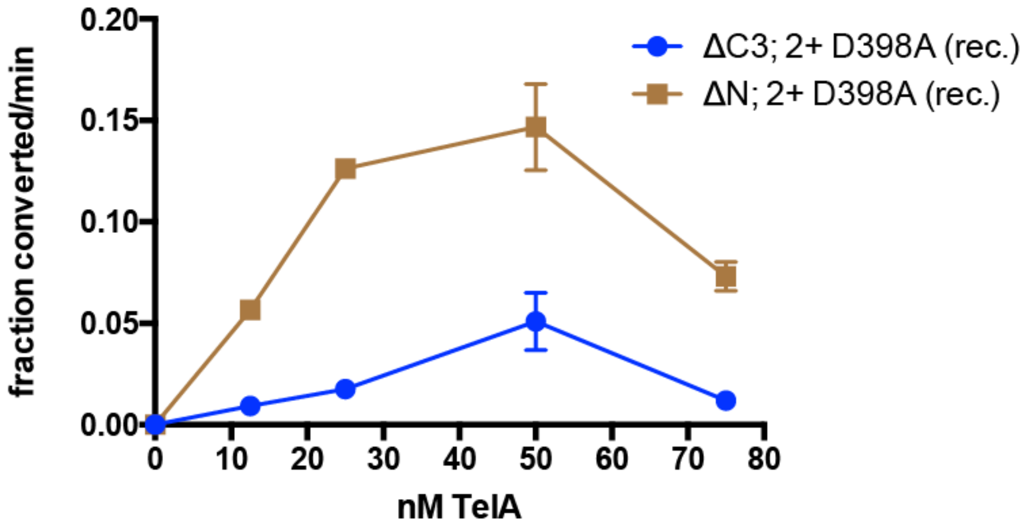

S5 Fig. TelA concentration optima for recombination by the hyperactive recombinase mutants. Shown is a summary graph of the initial rates of recombination (rec.) of the (ΔC3; D202RE337KD398A) **and** (ΔN; D202RE337KD398A) mutants plotted against TelA concentration. Both mutants were inactive for telomere resolution at all tested concentrations (not shown on plot). Shown are the mean and standard deviation of three independent trials of each experiment.

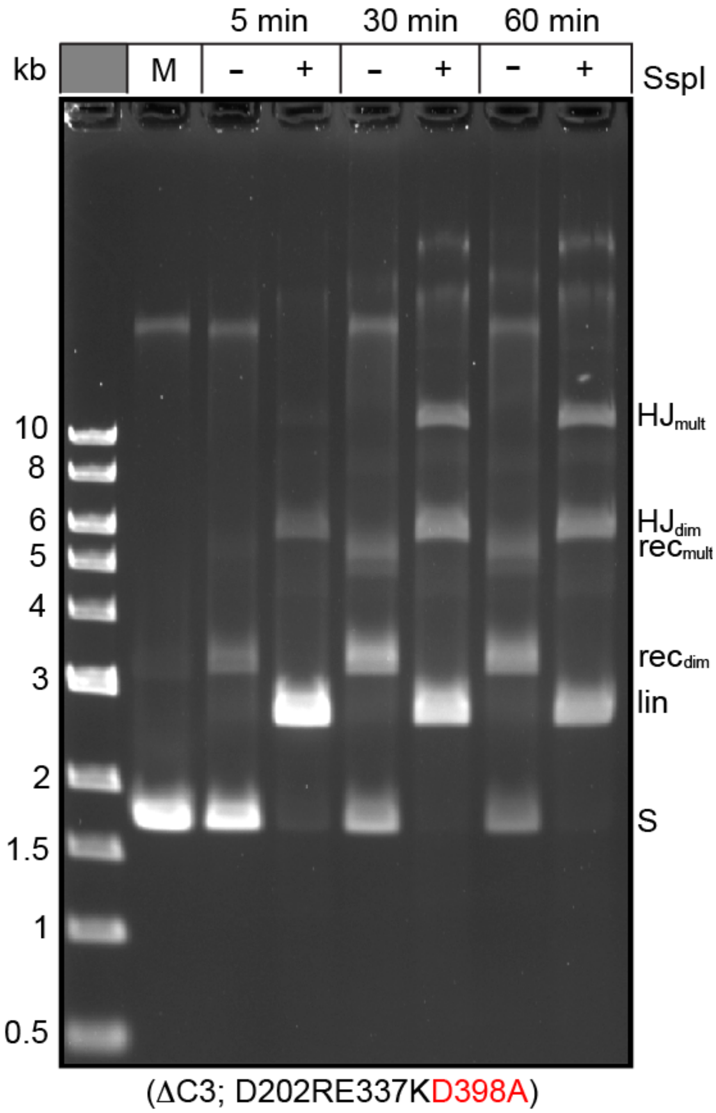

S6 Fig. Timecourse of the formation of recombinants and HJs.
Shown is a gel of a timecourse reaction of the (ΔC3; D202RE337KD398A) mutant reacted with negatively supercoiled pEKK494 (wild type substrate). Each timepoint was loaded -/+ SspI digestion to visualize recombinant dimers/multimers and HJ forms, respectively. S denotes supercoiled substrate; lin denotes SspI-linearized material (recombinants and unreacted substrate); rec_{dim} denotes a recombinant dimer; rec_{mult} denotes a recombinant multimer; HJ_{dim} denotes a HJ from a dimer; HJ_{mult} denotes a HJ multimer.

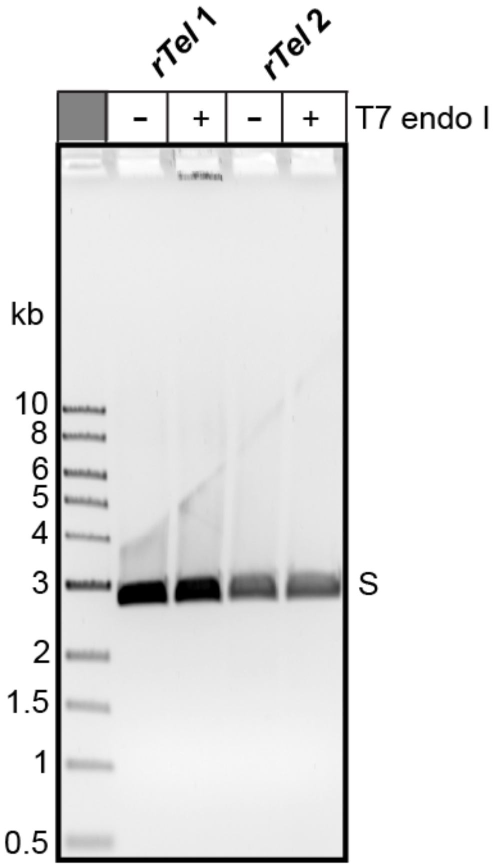

S7 Fig. Lack of background T7 endonuclease I cleavage of the substrate plasmids. Shown is a 0.8% agarose 1X TAE gel panel of SspI-linearized pEKK495 (*rTel* 1) or pEKK592 (*rTel* 2) with or without treatment with T7 endonuclease I at 30°C for 2 min.

S1 Table. Oligonucleotides used in this study.

Oligo name	Oligo sequence	Use
OGCB878	5′-CTGGGCCACAACAATAATGCGTTAGAAACAAGTCTTTCT-3′	Make D398A; ts; with WT and mutant parents
OGCB879	5'-AGAAAGACTTGTTTCTAACGCATTATTGTTGTGGCCCAG-3'	Make D398A; bs; with WT and mutant parents
OKBA19	5'-acagcagacgtgtcttggacgagaagtcgtc-3'	Make E337K; ts; WT parent
OKBA20	5'-gacgacttctcgtccaagacacgtctgctgt-3'	Make E337K; bs; WT parent
OKBA37	5'-aacacgacgagcttcgcggtacatagcagcgtcg-3'	Make D202R; ts; WT parent
OKBA38	5'-cgacgctgctatgtaccgcgaagctcgtcgtgtt-3'	Make D202R; bs; WT parent
OGCB962	5'-ATTGCGCCCGTAAGTTGAAAGGGGTAAGGATCC-3'	Make TelA (1-439); ts; WT parent
OGCB963	5'-GGATCCTTACCCCTTTCAACTTACGGGCGCAAT-3'	Make TelA (1-439); bs; WT parent
OKBA43	5'-aacacgacgagcttccctgtacatagcagcgtcg-3'	Make ∆C3; D202RE337K; ts
OKBA44	5'-cgacgctgctatgtacagggaagctcgtcgtgtt-3'	Make ∆C3; D202RE337K; bs
OGCB795	5′-CACCATGGCATATGGGAGTGGCGACCTCTATCGTTGAAAAG-3′	Make TelA (107-442); ts; WT parent
OGCB794	5′-ATACCGGATCCTTACCCCTTACGACTTACGGGCGC-3′	Make TelA

		(107-442); bs; WT parent
OKBA37	5'-aacacgacgagcttcgcggtacatagcagcgtcg-3'	Make ΔN; D202RE337K; ts; ΔN; E337K parent
OKBA38	5'-cgacgctgctatgtaccgcgaagctcgtcgtgtt-3'	Make ΔN; D202RE337K; bs; ΔN; E337K parent
OKBA27F	5′F-atcCCTCTAACCATTGCGCGATCGATCATAATAACAATATCA-3′	5’-fluorescein labeled ts half-site
OGCB871	5′-TGATATTGTTATTATGATCGATCGCGCAATGGTTAGAGG-3′	bs half-site
OKBA28F	5′-atcCCTCTAACCATTGCGCGATCGATCATAATAAC AATATCATGATATTGTTATTATGATCGATCGCGCAATGGTTAGAGG-3′	5’-fluorescein labeled hairpin telomere
OKBA29F	5′-atcGGAGATTGGTAACGCGCTAGCATGTATTATTGTTATAGT-3′	5’-fluorescein labeled ts mock half-site
OGCB914	5′-CCTCTAACCATTGCGCGATCGTACATAATAACAATATCA-3′	bs mock half-site
OKBA30F	5′-atcGGAGATTGGTAACGCGCTAGCATGTATTATTGTTATAGTA CTATAACAATAATACTAGCATGCGCGTTACCAATCTCC-3′	5’-fluorescein labeled mock hairpin telomere
OGCB951F	5′F-aaCTCTAACCATTGCGCGATCGATCATAATAACAATATCATGATATTG TTATTGTAATCGATCGCGGATCCCGGGCGTAGCCACGTAGGT-3′	5’-fluorescein labeled ts *rTel*
OGCB952	5′-gaACCTACGTGGCTACGCCCGGGATCCGCGATCGATTACAATAAC AATATCATGATATTGTTATTATGATCGATCGCGCAATGGTTAGAG-3′	bs *rTel*
OKBA45F	5′F-aaGAGATTGGTAACGCGCTAGCTAGTATTATTGTTATAGTACTATAACA ATAACATTAGCTAGCGCCTAGGGCCCGCATCGGTGCATCCA-3′	5’-fluorescein labeled ts

		mock *rTel*
OKBA46	5′-gaTGGATGCACCGATGCGGGCCCTAGGCGCTAGCTAATGTTATTGTTATA<br>GTACTATAACAATAATACTAGCTAGCGTTACCAATCTC-3′	bs mock *rTel*
OGCB951	5′-aaCTCTAACCATTGCGCGATCGATCATAATAACAATATCATGATATTGTT<br>ATTGTAATCGATCGCGGATCCCGGGCGTAGCCACGTAGGT-3′	ts *rTel*
OKBA45	5′-aaGAGATTGGTAACGCGCTAGCTAGTATTATTGTTATAGTACTATAACAAT<br>AACATTAGCTAGCGCCTAGGGCCCGCATCGGTGCATCCA-3′	ts mock *rTel*
OGCB984	5′-gatcCTCTAACCATTGCGCGATCGATCATAATAACAATACCATGATATTGT<br>TATTGTAATCGATCGCGGATCCCGGGCGTAGCCACGTAG-3′	ts mutant *rTel* for BamHI-HindIII cloning into pUC19
OGCB985	5′-agctCTACGTGGCTACGCCCGGGATCCGCGATCGATTACAATAACAATATC<br>ATGGTATTGTTATTATGATCGATCGCGCAATGGTTAGAG-3′	bs mutant *rTel* for BamHI-HindIII cloning into pUC19
OKBA61	5′-gatcCCTCTAACCATTGCGCGATCGATCATAATAACAATATCA-3′	ts half-site for BamHI-HindIII cloning into pUC19
OKBA62	5′-agctTGATATTGTTATTATGATCGATCGCGCAATGGTTAGAGG-3′	bs half-site for BamHI-HindIII cloning into pUC19
OKBA63	5′-gatcGGAGATTGGTAACGCGCTAGCATGTATTATTGTTATAGT-3′	ts mock half-site for BamHI-HindIII cloning into pUC19
OKBA64	5′-agctACTATAACAATAATACATGCTAGCGCGTTACCAATCTCC-3′	bs mock half-site for BamHI-HindIII cloning into pUC19
OKBA65	5′-gatcGAGATTGGTAACGCGCTAGCTAGTATTATTGTTATAGTACTATAACAAT<br>AACATTAGCTAGCGCCTAGGGCCGCATCGGTGCATCCA-3′	ts mock *rTel* for BamHI-HindIII cloning into

		pUC19
OKBA66	5’-agctTGGATGCACCGATGCGGGCCCTAGGCGCTAGCTAATGTTATTGTTATAG TACTATAACAATAATACTAGCTAGCGCGTTACCAATCTC-3’	bs mock *rTel* for BamHI-HindIII cloning into pUC19
OKBA67	5'-gatcCTCTAACCATTGCGCGATCGATCATAATAACAATACCTTGATATTGTTA TTGTAATCGATCGCGGATCCCGGCGTAGCCACGTAG-3’	ts asymmetric *rTel* for BamHI-HindIII cloning into pUC19 pEKK495
OKBA68	5’-agctCTACGTGGCTACGCCCGGGATCCGCGATCGATTACAATAACAATATCAA GGTATTGTTATTATGATCGATCGCGCAATGGTTAGAG-3’	bs asymmetric *rTel* for BamHI-HindIII cloning into pUC19 pEKK495
OKBA67F	5'F- aaCTCTAACCATTGCGCGATCGATCATAATAACAATACCTTGATATTGTTATTGTA ATCGATCGCGGATCCCGGGCGTAGCCACGTAG-3’	ts asymmetric *rTel with* 5’ fluorescein
OKBA68F	5’F-aaCTACGTGGCTACGCCCGGGATCCGCGATCGATTACAATAACAATATCAAG GTATTGTTATTATGATCGATCGCCAATGGTTAGAG-3’	bs asymmetric *rTel with* 5’ fluorescein
OKBA69	**5'-gatcCTCTAACCATTGCGCGATCGATCATAATAACAATACATTGATATTGTTA TTGTAATCGATCGCGGATCCCGGGCGTAGCCACGTAG-3’**	ts asymmetric *rTel* for BamHI-HindIII cloning into pUC19 pEKK592
OKBA70	5’-agctCTACGTGGCTACGCCCGGGATCCGCGATCGATTACAATAACAATATCAA TGTATTGTTATTATGATCGATCGCGCAATGGTTAGAG-3’	bs asymmetric *rTel* for BamHI-HindIII cloning into

		pUC19<br>pEKK592

Table S2. Induction/expression conditions for TelA mutants.

Strain #	Mutant	Induction/expression conditions
EKK395	Wild type	
EKK452	D398A	
EKK572	ΔN; D398A	0.25 mM IPTG induction at 24°C O/N (1L)
EKK573	ΔC3; D202RE337KD398A	0.25 mM IPTG induction at 24°C O/N (1L)
EKK586	ΔN; D202RE337KD398A	0.5 mM IPTG induction at 24°C for 6 h (2L)

Supplemental Material and Methods

Protein-protein crosslinking assessment of the DNA-dependence of TelA oligomerization.

The ability of TelA to oligomerize was assessed by protein-protein crosslinking with 0.005% glutaraldehyde. Incubation of 74 nM with the indicated TelA variants was performed in a buffer containing 25 mM HEPES (pH 7.6), 1 mM DTT, 2 mM $MgCl_2$ and 50 mM potassium glutamate with or without the addition of negatively supercoiled 10 μg/mL pEKK494 at 0°C for 20 min prior to the addition of protein crosslinker. Crosslinking reactions were conducted at room temperature for 5 min and then the reactions were terminated by addition of protein SDS load dye to a 1X concentration (1X load dye contains 50 mM Tris [pH 6.8], 2% [w/v] SDS, 0.1%[w/v] bromophenol blue, 5% β-mercaptoethanol and 10% [w/v] glycerol). TelA oligomerization status was visualized by application of the reactions, after protein denaturation at 95°C for 5 min, to a 5/10% SDS-PAGE gel run at 20V/cm for 2 h. The resulting gels were transferred to PVDF membranes and western blotted with a mouse primary antibody directed against the His-tag (Millipore; 1/10, 000 dilution). The signal was amplified by a goat anti-mouse secondary antibody (BioRad; 1/10, 000 dilution) and the final result was visualized by chemiluminescence on a BioRad GelDoc system.
